# Genome-Wide Analysis of Coding and Long Non-Coding RNAs Involved in Cuticular Wax Biosynthesis in Cabbage (*Brassica oleracea* L. var. *capitata*)

**DOI:** 10.3390/ijms20112820

**Published:** 2019-06-10

**Authors:** Xiaowei Zhu, Xiang Tai, Yunying Ren, Jinxiu Chen, Tianyue Bo

**Affiliations:** Horticulture Research Institute, Shanghai Academy of Agricultural Sciences, Shanghai Key Lab of Protected Horticultural Technology, Shanghai 201403, China; xiaoweizhusaas@163.com (X.Z.); 555666tx@163.com (X.T.); yy11@saas.sh.cn (Y.R.)

**Keywords:** cabbage, *nwgl* phenotype, fine mapping, cuticular wax biosynthesis, RNA-seq, differential expressed genes, lncRNAs

## Abstract

Cuticular wax is a mixture of very long chain fatty acids (VLCFAs) and their derivatives, which determines vital roles for plant growth. In cabbage, the cuticular wax content of leaf blades is an important trait influencing morphological features of the head. Understanding the molecular basis of cuticular wax biosynthesis can help breeders develop high quality cabbage varieties. Here, we characterize a cabbage *non-wax glossy* (*nwgl*) plant, which exhibits glossy green phenotype. Cryo-scanning electron microscope analysis showed abnormal wax crystals on the leaf surfaces of *nwgl* plants. Cuticular wax composition analyzed by GC-MS displayed severely decreased in total wax loads, and individual wax components in *nwgl* leaves. We delimited the *NWGL* locus into a 99-kb interval between the at004 marker and the end of chromosome C08 through fine mapping. By high-throughput RNA sequencing, we identified 1247 differentially expressed genes (DEGs) and 148 differentially expressed lncRNAs in *nwgl* leaves relative to the wild-type. Gene Ontology (GO) and Kyoto Encyclopedia of Genes and Genomes (KEGG) pathway enrichment analysis revealed that the DEGs and *cis*-regulated target genes for differentially expressed lncRNAs were significantly enriched in wax and lipid biosynthetic or metabolic processes. Our results provide the novel foundation to explore the complex molecular basis of cuticular wax biosynthesis.

## 1. Introduction

The aerial surface of land plants is covered with a hydrophobic cuticle layer, which is composed of two major lipid components, the cutin polyester and the non-polymerized cuticular waxes. Cuticular waxes are predominantly composed of very long chain fatty acids (VLCFAs) and their derivatives, including aldehydes, alcohols, alkanes, ketones, and esters [1,2]. The biosynthesis of cuticular wax begins with de novo synthesized C16 and C18 fatty acids, which are then elongated into VLCFAs by a fatty acid elongase (FAE) complex, consisting of β-ketoacyl-CoA synthase, β-ketoacyl-CoA reductase, β-hydroxyacyl-CoA dehydratase, and enoyl-CoA reductase. The formed VLCFAs are further converted into various derivatives via two independent pathways, the alkane-forming and alcohol-forming pathways [3,4]. Cuticular wax protects plants against numerous biotic and abiotic stresses, such as pathogen infection and non-stomatal water loss [5,6]. It also plays important roles in normal plant developmental processes, including the pollen–pistil interaction [7]. Therefore, identifying plants with cuticular wax deficiency and elucidating the regulatory mechanisms controlling cuticular wax biosynthesis will be beneficial for improving agricultural crops. 

To date, many genes associated with cuticular wax biosynthesis pathway have been isolated and characterized in different plant species. For example, *CER1* encodes an alkane-forming enzyme and may physically interact with CER3 and cytochrome b5 isoforms (CYTB5s) to catalyze the synthesis of very long chain alkanes [8,9]. CER4 is an alcohol-forming fatty acyl-coenzyme A reductase (FAR) in the synthesis of primary alcohols [10]. In addition, several APETALA2/ETHYLENE-RESPONSIVE FACTOR (AP2/EFR) type transcription factors have also been reported to be involved in cuticular wax biosynthesis through regulating the expression of wax biosynthesis genes [11,12,13,14,15,16,17]. For instance, WRINKLED4 (WRI4) activates the expression of *LACS1*, *KCR1*, *PAS2*, *ECR*, and *WSD1* by direct binding to their promoters [16]. 

In recent years, several RNA transcripts with no protein coding potential have been reported that have a key role in regulation of cuticular wax biosynthesis. These noncoding RNAs include some small RNA species, such as small interfering RNA (siRNA) and microRNA (miRNA), mediated silencing transcription of the wax biosynthetic genes [18,19,20,21,22]. Cuticular wax biosynthesis also involves long noncoding RNAs (lncRNAs). LncRNAs are a class of non-coding RNA transcripts with a length of more than 200 nt. In wheat, *INHIBITOROFWAX1* (*Iw1*) and its homolog *Iw2* serve as miRNA precursors that produce an miRNA, miRW1, which represses the expression of the putative carboxylesterase genes that are necessary for β-diketone deposition in wheat [22].

For cabbage, several cuticular wax deficiency mutants have been reported. The phenotype of 10Q-961 and g21-3 mutants is controlled by a same recessive locus, which was finely mapped to chromosome C08 through classical linkage analysis [23,24]. A dominant 10Q-974gl mutant gene *BoGL1* was also fine mapped to chromosome C08 [25]. Moreover, both the *BoWAX1* and *Cgl2* genes were mapped to chromosome C01 [26,27]. In addition, the *Cgl2* gene in cabbage had been cloned and could rescue the phenotype of the *Arabidopsis cer4* mutant [27]. However, the cuticular wax biosynthesis genes have not been fully explored in cabbage.

In this study, we report the phenotypic and characterization of the *non-wax glossy* (*nwgl*) plant characterized by a glossy appearance of the cabbage. The *nwgl* plants exhibited a marked reduction in amounts of total wax and changes in wax components. We performed fine mapping and delimited the *NWGL* gene responsible for the *nwgl* phenotype to the end of chromosome C08. By conducting RNA-seq, we identified differential expressed genes (DEGs) and lncRNAs in leaf tissues of *nwgl* and wild-type plants. Moreover, we predicted both *cis-* and *trans-* regulatory target genes for the differential expressed lncRNAs. Functional analysis of the DEGs and *cis*-regulated targets genes for differential expressed lncRNAs revealed significantly enriched categories and pathways related with wax and lipid biosynthesis and/or metabolism, providing novel implication underlying cuticular wax biosynthesis in cabbage.

## 2. Results

### 2.1. Characterization of the Plant Exhibiting nwgl Phenotype

**Figure 1:**
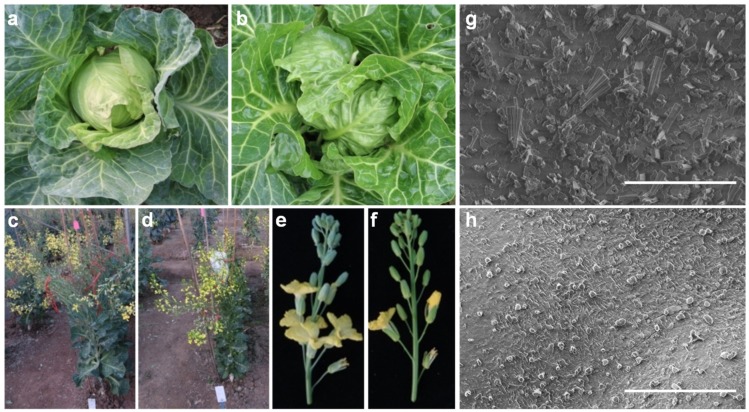
Phenotypes of wild-type and *nwgl* plants. The leaf appearance of wild-type (**a**) and *nwgl* plants (**b**) at the head harvesting stage. Wild-type (**c**) and *nwgl* (**d**) plants at the flowering stage. (**e**) Wild-type flowers. (**f**) *nwgl* flowers. Cuticular wax crystals formed on adaxial surface of the wild-type (**g**) and *nwgl* (**h**) leaf blades visualized by cryo-scanning electron microscopy. Bar = 10 μm in (**g**) and (**h**).

The plant exhibiting *nwgl* phenotype was identified from the inbred line G287. The prominent phenotype of *nwgl* plants was their altered wax coatings, resulting in light green leaves, stems, and flower buds (Figure 1a–f). The plant was designated *nwgl* based on its non-wax and glossy appearance. Cryo-scanning electron microscopy analysis revealed that the cuboid wax crystals were distributed densely on the leaf surface of the wild-type, whereas only fewer granular wax crystals were deposited on the leaf surface of *nwgl* plants (Figure 1g,h). Therefore, these results indicate that the *nwgl* mutation might cause abnormalities in specific components of the cuticular wax of cabbage plants. 

### 2.2. Cuticular Wax Composition of nwgl Plants

**Figure 2:**
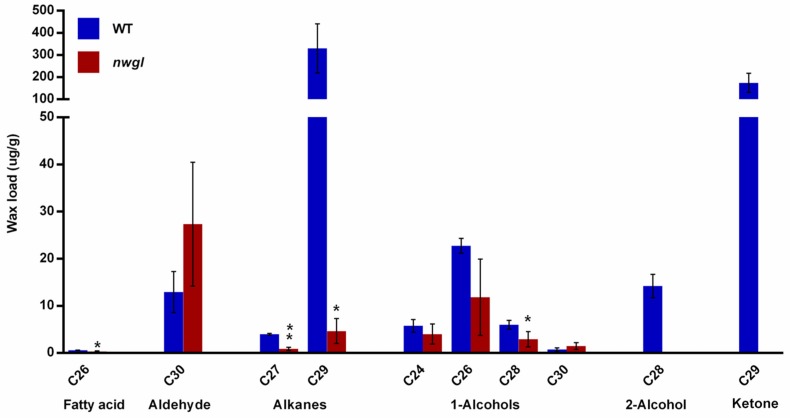
Cuticular wax composition in leaves from wild-type and *nwgl* plants. Error bars indicate SD (*n* = 3). * *p* < 0.05; ** *p* < 0.01.

To investigate why the plants exhibiting *nwgl* phenotype had a significant depletion in wax crystals, we examined the content and the constitution of cuticular waxes from leaf blades of *nwgl* and wild-type plants by gas chromatography-mass spectrometry (GC-MS). Compared with wild-type plants, the total amount of all wax loads on *nwgl* leaf blades was reduced by 90.7% (Table 1). The contents of VLCFA, alkanes, and primary alcohols decreased by 45.8%, 98.3%, and 42.5% in *nwgl* leaf blades, respectively (Table 1). The secondary alcohol and ketone were undetectable in *nwgl* leaf blades (Table 1). However, the content of aldehyde exhibited slightly increased in *nwgl* leaf blades (Table 1). Further analysis revealed significantly reduced contents of C26 VLCFA, C27 and C29 alkanes, and C28 primary alcohol. Meanwhile, neither C28 secondary alcohol nor C29 ketone was detected in *nwgl* leaf blades (Figure 2). Taken together, these findings suggest that the reduced wax crystals in *nwgl* leaf blades result from decreased levels of VLCFAs and their individual derivatives. 

### 2.3. Fine Mapping of NWGL

**Figure 3:**
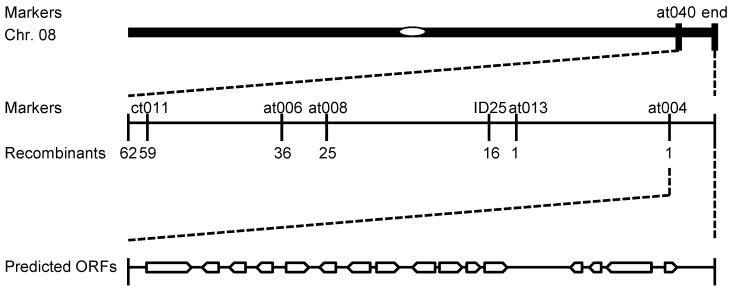
Fine mapping of *NWGL*. The *NWGL* locus was mapped onto chromosome C08. Molecular markers and ORFs in the mapping region are shown.

**Figure 4:**
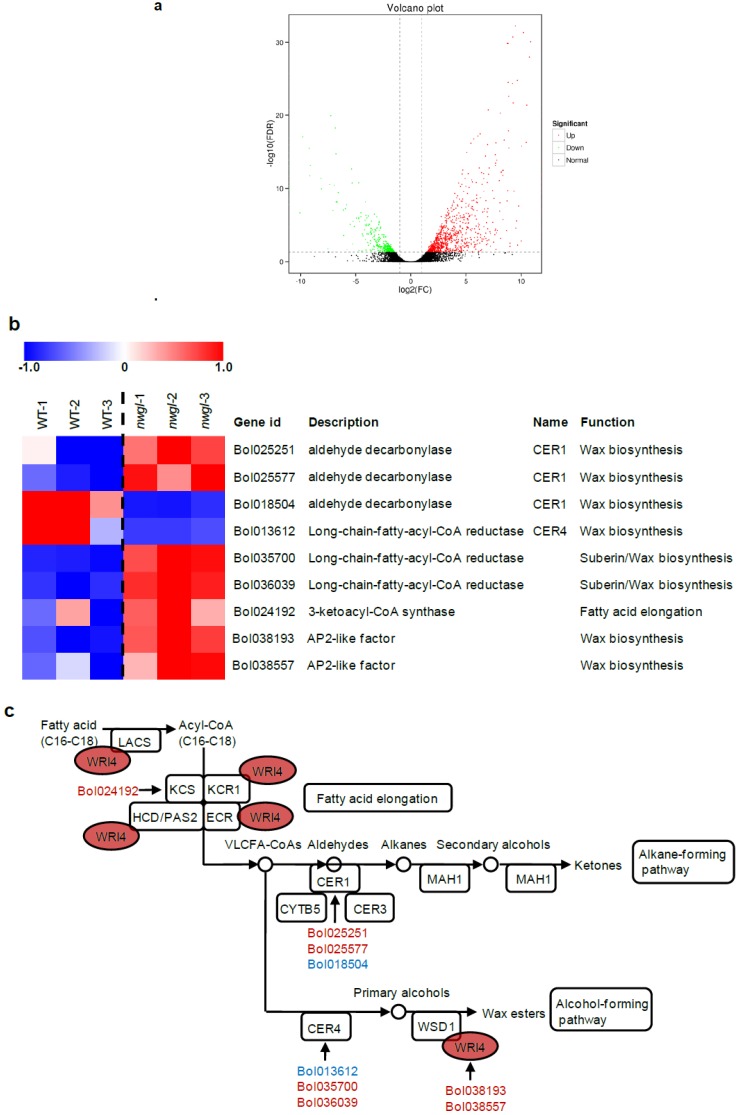
Analysis of DEGs in the RNA-seq libraries. (**a**) Volcano plot showing significant DEGs. Red and green dots represent up and down-regulated DEGs, respectively (FDR <0.05). Black dots are genes that were not differently expressed. (**b**) Gene expression of DEGs involved in wax biosynthesis. The data are shown in base 2 logarithmic form (log2 FPKM). Three biological replicates of wild-type (WT) and *nwgl* leaves are exhibited. (**c**) Simplified pathways for wax biosynthesis. LACS, long chain acyl-CoA synthetase; KCS, β-ketoacyl-CoA synthase; KCR, β-ketoacyl-CoA reductase; HCD/PAS2, β-hydroxyacyl-CoA dehydratase; ECR, enoyl-CoA reductase; CER, ECERIFERUM; CYTB5, cytochrome b5 isoform; MAH, mid-chain alkane hydroxylase; WSD, wax synthase/diacylglycerol acyltransferase; WRI4, WRINKLED4. Down-regulated and up-regulated genes in *nwgl* vs. wild-type plants are marked in green and red color, respectively.

Genetic analysis showed that the *nwgl* phenotype is a dominant mutation controlled by a single gene, with one quarter of the F_2_ progeny displaying a normal waxy appearance (208:61, χ^2^ = 0.67, *p* > 0.05; 270:91, χ^2^ = 0.04, *p* > 0.05). To isolate the *NWGL* gene, we generated two F_2_ mapping populations by crossing plants exhibiting *nwgl* phenotype with two wild-type inbred lines G306 and G274, respectively. The *NWGL* locus was mapped preliminarily between simple sequence repeat (SSR) marker at 040 and the end of chromosome C08 (Figure 3). Using newly developed InDel and SSR markers, the *NWGL* gene was fine-mapped into a 99-kb DNA segment between marker at004 and the end of chromosome C08 (Figure 3). There were 16 putative open reading frames (ORFs) within the 99-kb genomic region according to the cabbage gene annotation database (www.ocri-genomics.org/bolbase, accessed on: 9 June 2019) (Figure 3). Except for *Bol018503* and *Bol018504*, the functions of these genes are either unknown or do not involve in cuticular wax biosynthesis (Table S1). *Bol018503* encodes a CER1-L1 homolog, which belongs to fatty acid hydroxylase superfamily in Arabidopsis, and *Bol018504* encodes an aldehyde decarbonylase (CER1), which is known to promote wax very long chain alkane biosynthesis in various plant species [8,9,28,29]. To define which of these carries the *nwgl* mutation, we sequenced the two candidate genes, including their 5′ and 3′ flanking sequences, but no DNA sequence alteration was found. However, examination of gene expression of all the 16 ORFs within the fine mapping region only showed a down-regulation of *Bol018504* in *nwgl* leaves but not in the wild-type, indicating that the decreased expression of *Bol018504* might cause the *nwgl* phenotypes (Figure 4b, Table S2).This speculation was in accordance with a recent study, in which the same expression abundance decrease of *Bol018504* was identified in an investigation of a dominant glossy green cabbage mutant 10Q-974gl [25]. 

These results suggest that the *Bol018504* might be regulated by the *NWGL* locus or other mechanisms, such as an epigenetic manner. We further investigated the DNA methylation level of *Bol018504* by employing three methylation sensitive restriction enzymes. For *Mcr*BC-PCR analysis, we found that the middle region of *Bol018504* corresponding to the fragment “C” (+905 to +1615) was unmethylated in the wild-type but slightly methylated in *nwgl* plants (Figure S1a,b).The *Hpa*Ⅱ, *Msp*Ⅰ-PCR analysis was used to detect the methylation level of CCGG sites within fragments “A” (−1113 to −408), “B”(+189 to +1069), and “C”(+905 to +1615). The results indicated that the C of the CCGG sites within fragments “B”(+189 to +1069) and “C”(+905 to +1615) might be hypermethylated in *nwgl* plants compared with the wild-type (Figure S1a,c). 

### 2.4. RNA-seq Analysis of Plants Exhibitingnwgl Phenotype

To further explore the global impacts of the *nwgl* mutation which could reduce cuticular wax contents in plants exhibiting *nwgl* phenotype, we conducted a transcriptome comparison of *nwgl* and wild-type leaves by high-throughput RNA sequencing (RNA-seq). In total, 1247 protein coding genes were differentially expressed in *nwgl* leaves relative to wild-type, including 913 up-regulated genes and 334 down-regulated genes (Figure 4a, Table S2). 

We then performed Gene Ontology (GO) analysis to assess the function of the DEGs. Notably, the down-regulated genes most significantly enriched were in the wax biosynthetic process (GO: 0010025), while the up-regulated genes most significantly enriched were in response to chitin (GO: 0010200) (Tables S3 and S4). In addition, a significant fraction of up-regulated genes were involved in lipid-related GO categories, including lipid localization (GO: 0010876), positive regulation of fatty acid metabolic/biosynthetic process (GO: 0045923 and GO: 0045723), glycerolipid metabolic process (GO: 0046486), neutral lipid metabolic process (GO: 0006638) and sphingolipid catabolic process (GO: 0030149) (Table S4), suggesting that *NWGL* might be involved in wax biosynthesis through regulating the expression of lipid-related genes. 

To further understand the metabolic pathways associated with *NWGL*, the DEGs were mapped to the KEGG database to analyze the enriched pathways. There were nine pathways highly enriched for down-regulated genes, and the most significantly enriched encoded proteins were involved in ‘tyrosine metabolism’ (KEGG ID: ko00350) (Table S5). The ‘cutin, suberine and wax biosynthesis’ (KEGG ID: ko00073), ‘linoleic acid metabolism’ (KEGG ID: ko00591), and ‘sphingolipid metabolism’ (KEGG ID: ko00600) were significantly enriched pathways in up-regulated genes (Table S6), supporting the notion that NWGL functions in wax and lipid metabolism.

When focusing on the wax biosynthetic process, nine genes showed an altered expression in *nwgl* leaves relative to the wild-type. Only two genes associated with wax biosynthesis were down-regulated in *nwgl* leaves (genes encoding CER1 (*Bol018504*) and CER4 (*Bol013612*)), while seven genes were up-regulated: genes encoding another two CER1 proteins (*Bol025251* and *Bol025577*), two Long-chain-fatty-acyl-CoA reductases (*Bol035700* and *Bol036039*), one 3-ketoacyl-CoA synthase (*Bol024192*) and two AP2-like factors (*Bol038193* and *Bol038557*) (Figure 4b,c).

### 2.5. Identification and Characterization of lncRNAs in Cabbage

**Figure 5:**
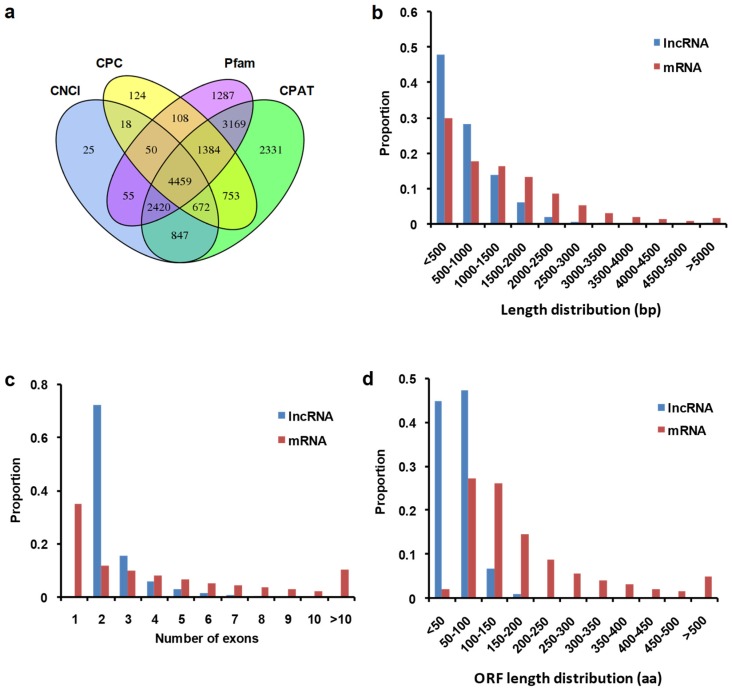
Characteristics of lncRNAs identified in cabbage. (**a**) The 4459 lncRNAs in cabbage were identified by an intersection of results obtained via three softwares CNCI (Coding-Non-Coding Index), CPC (Coding Potential Calculator), CPAT (Coding Potential Assessment Tool) and a protein database Pfam analysis. (**b**) Length distribution (base pair, bp) of lncRNAs and mRNAs. (**c**) Exon number in lncRNAs and mRNAs. (**d**) ORF length in amino acids (aa) of lncRNAs and mRNAs.

To screen out potential lncRNAs involved in regulating wax biosynthesis, we thoroughly analyzed the cabbage noncoding transcriptome and identified 4459 putative lncRNAs (Figure 5a, Table S7). The 4459 lncRNA sequences were compared with mRNAs obtained in this study. These lncRNAs were shorter than mRNAs, 76.3% of which being <1000 bp in length (Figure 5b). Most lncRNAs (88.0%) had two or three exons in their transcripts and were fewer than protein-coding transcripts (Figure 5c). Interestingly enough, the two features of lncRNAs in cabbage, shorter lengths and fewer exons, have also been detected in other *Brassica* species, including Chinese cabbage, rapeseed, kale, and non-heading Chinese cabbage [30,31,32]. Moreover, lncRNAs also had shorter ORFs compared with mRNAs, with approximately 92.2% at <100 amino acids (Figure 5d). 

### 2.6. Identification of Differentially Expressed lncRNAs between nwgl and Wild-Type Plants

**Figure 6:**
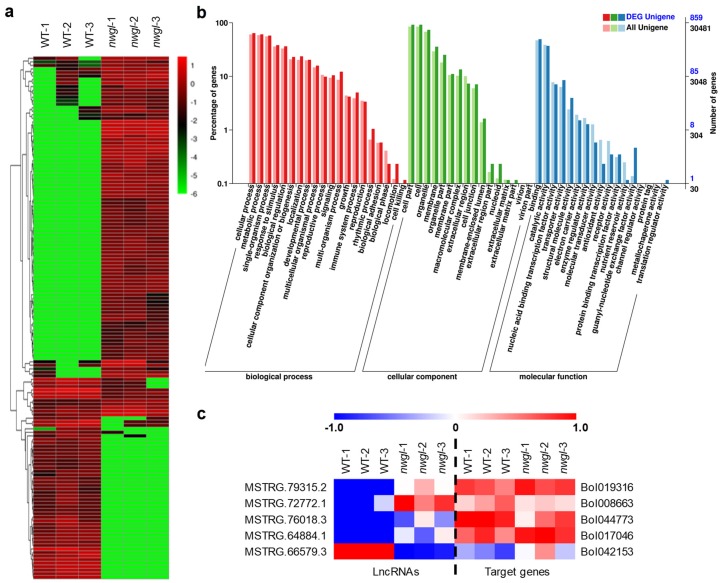
Differentially expressed lncRNAs in *nwgl* vs. wild-type plants. (**a**) Hierarchical clustering analysis of the differentially expressed lncRNAs. The data are expressed in base 10 logarithmic form (log10 FPKM+0.000001). (**b**) GO classification of 906 *cis*-regulated target genes of differentially expressed lncRNAs. (**c**) The co-expression differentially expressed lncRNAs and target genes involved in lipid-related processes. The data are exhibited in base 2 logarithmic form (log2 FPKM). Three biological replicates of wild-type (WT) and *nwgl* plants are shown.

A total of 148 lncRNAs were found to be significantly differentially expressed (Figure 6a, Table S8). In detail, 96 lncRNAs in *nwgl* leaves were up-regulated and 52 were down-regulated compared with the wild-type (Figure 6a, Table S8). LncRNAs have been shown to regulate protein coding gene expression via *cis*- and *trans*-action [33]. In total, 929 annotated target genes for 148 differentially expressed lncRNAs were predicted, of which 906 were *cis*-regulatory and 23 were *trans*-regulatory (Tables S9 and S10). Gene Ontology (GO) terms were identified for the 906 *cis*-regulated target genes (Figure 6b). The top enriched GO terms in the biological process, cellular component, and molecular function categories were organic cyclic compound biosynthetic process (GO: 1901362), nucleus (GO: 0005634), and protein binding (GO: 0005515), respectively (Table S11). In addition, the GO term glycerolipid metabolic process (GO: 0046486) was highly enriched (Table S11). Moreover, the 906 *cis*-regulated target genes were significantly enriched in KEGG pathways, consisting of the pentose phosphate pathway (KEGG ID: ko00030), endocytosis (KEGG ID: ko04144), ribosome (KEGG ID: ko03010), glycolysis/gluconeogenesis (KEGG ID: ko00010), degradation of aromatic compounds (KEGG ID: ko01220), and oxidative phosphorylation (KEGG ID: ko00190) (Table S12). 

To further analyze the function of differentially expressed lncRNAs, we investigated the co-expression correlation between differentially expressed lncRNAs and their predicted target genes. A total of 206 interaction relationships (111 positive and 95 negative correlations) were discovered between 97 lncRNAs and 163 target genes (Table S13). Notably, some of the correlated target genes were involved in lipid-related processes, including fatty acid metabolic process, fatty acid beta-oxidation, fatty acid degradation, and fatty acid homeostasis (Figure 6c).

## 3. Discussion

The content of cuticular waxes is an important agronomic trait for cabbage breeding. However, there are only a few reports regarding wax biosynthesis in cabbage [23,24,25,26,27]. The phenotype and characterization of the *nwgl* plants reported here showed that *NWGL* is a factor for cuticular wax biosynthesis in cabbage. The *nwgl* leaf blades exhibited decreased contents of VLCFA, alkanes and primary alcohols, and the abortion of secondary alcohol and ketone (Table 1), indicating that NWGL acted as an major regulator for VLCFA elongation and the two distinct pathways, the alkane-forming and alcohol-forming pathways. The involvement of NWGL in the synthesis of various wax compounds was reconfirmed by the RNA-seq analysis. Firstly, GO and KEGG pathway analysis showed that DEGs between plants exhibiting *nwgl* phenotype and the wild-type were enriched in categories related to wax and lipid synthesis/metabolism (Tables S3–S6). Secondly, nine DEGs which encode proteins involved in VLCFA production, alkane-forming and alcohol-forming pathways of wax biosynthesis are regulated by NWGL (Figure 4c). Thirdly, five of the target genes that were found to be strongly co-expressed with the differentially expressed lncRNAs were annotated to be involved in lipid-related processes (Figure 6c). Collectively, our data suggest that NWGL is involved in multiple wax biosynthetic processes, from upstream fatty acid synthesis to subsequent wax production. 

Our results demonstrated that the glossy phenotype of the *nwgl* plants is determined by a single dominant locus within approximately 99-kb between at004 and the end of chromosome C08 (Figure 3). Therefore, these results indicated that the *NWGL* gene may be an allele of the previously studied *BoGL1* gene [25]. Unfortunately, we failed to identify DNA lesion of *Bol018504* in *nwgl* plants, except its reduced expression abundance and altered DNA methylation level, which was located in the fine mapping region and encodes an alkane-forming enzyme (BoCER1). Previous studies have reported that the *Arabidopsis thaliana* CER1 protein is an essential element of wax alkane synthesis [8,9]. The Arabidopsis *cer1* mutant exhibited a dramatic decrease in alkanes and nearly depleted of secondary alcohols and ketones, accompanied by a slight increase in aldehyde content as compared to wild-type plants [8,34,35,36]. Interestingly, our GC-MS analyses found significant reductions in total wax in *nwgl* leaf blades, especially for C27 and C29 alkanes, and the undetected C28 secondary alcohol and C29 ketone, indicating that *Bol018504* played a critical role in NWGL-regulated cuticular wax biosynthesis. However, we cannot rule out the possibility that other unknown genes within the fine mapping region may be responsible for *NWGL*, which may prove another interesting area for further investigation. For instance, the VLCFA component, which cannot be catalyzed by CER1, also decreased significantly in *nwgl* leaf blades.

The interaction of *NWGL* with other genes was investigated via transcriptome analysis. Based on our RNA-seq data, the expression of nine genes, whose homologs in *Arabidopsis thaliana* are key factors involved in wax biosynthesis, was down-regulated or up-regulated in *nwgl* leaves [8,10,16,37,38]. Except for *Bol018504*, the expression of *Bol013612* was down-regulated in *nwgl* leaves. *Bol013612*, which encodes fatty acyl-CoA reductase, is homologous to *Arabidopsis thaliana CER4* [10]. The wax components of the cabbage *Bol013612* loss-of-function mutant were lacking in primary alcohols and wax esters [27]. Moreover, the *nwgl* leaf blades showed significant reduction in C28 primary alcohol, suggesting that the effect of *NWGL* on wax biosynthesis might require *Bol013612* at the mRNA level. However, the expression levels of five genes, which encode one 3-ketoacyl-CoA synthase (*Bol024192*), another two CER1 proteins (*Bol025251* and *Bol025577*), and two fatty-acyl-CoA reductases (*Bol035700* and *Bol036039*), were up-regulated in *nwgl* leaves. These results indicated that the altering wax amount in the *nwgl* mutant resulted in feedback, causing up-regulation of some genes in fatty acid elongation, alkane-forming, and alcohol-forming pathways of wax biosynthesis. In addition, transcriptional control is considered to be the major mechanism for determining the total wax deposition in *Arabidopsis thaliana* [2]. The remaining two up-regulated genes—*Bol038193* and *Bol038557*—in *nwgl* leaves, which encode AP2-like factors, are 81.2% and 88.0% homologous to *Arabidopsis thaliana* WRI4 transcription factor, respectively. WRI4 is a transcriptional activator and functions in the up-regulation of cuticular wax biosynthesis in Arabidopsis stems [16]. Therefore, these findings suggest that the up-regulated genes in *nwgl* leaves might be controlled by NWGL through WRI4 transcription factor. 

Although the number of known plant lncRNAs is increasing dramatically, the identification of lncRNAs as regulators in cuticular wax biosynthesis in plants is still lacking [22,30,31,32,39,40,41,42,43,44,45,46]. In this study, we identified 148 differentially expressed lncRNAs between plants exhibiting *nwgl* phenotype and wild-type (Figure 6a, Table S8), revealing specific responses of the noncoding transcriptome to cuticular wax loads reduction. The fact that these lncRNAs changed expression pattern in *nwgl* leaves suggested that at least some of them have regulatory roles in wax biosynthesis. LncRNAs can regulate gene expression by *cis-* and *trans-*acting [33]. We predicted the *cis-*regulated and *trans-*regulated target genes of the differentially expressed lncRNAs and performed a co-expression analysis between them to explore the putative regulatory functions of the lncRNAs. Five of the target genes, which were found to be strongly co-expressed with the differentially expressed lncRNAs, were annotated to be involved in lipid-related processes. Unfortunately, we did not find any target genes of the differentially expressed lncRNAs directly involved in cuticular wax biosynthesis. It would be difficult, but worthwhile, to investigate the exact roles of these differentially expressed lncRNAs in cuticular wax biosynthesis. 

In summary, we fine-mapped the *NWGL* locus underlying the non-wax glossy phenotype in cabbage. Through genome-wide transcriptome studies, we revealed that many protein-coding genes involved in wax biosynthesis were regulated by the *NWGL* gene. Perhaps the most striking finding by this work was genome-wide identification of a set of lncRNAs differentially expressed in plants exhibiting *nwgl* phenotype, and may reveal a more sophisticated molecular regulation mechanism of cuticular wax biosynthesis in cabbage.

## 4. Materials and Methods

### 4.1. Plant Materials

The cabbage (*Brassica oleracea* L. var. *capitata*) plant exhibiting (*non-wax glossy*) *nwgl* phenotype was derived from inbred line G287. Two F_2_ mapping populations were generated by crossing the plant exhibiting *nwgl* phenotype with inbred line G306 or G274, respectively. Cabbage plants were cultivated in the experimental field at the Shanghai Academy of Agricultural Sciences (Shanghai, China) during the natural growing season.

### 4.2. Cryo-scanning Electron Microscopy (Cryo-SEM)

Fresh leaf samples from *nwgl* and wild-type plants were fixed in specimen holders of a Hitachi S4800 cryo-transfer system (Hitachi, Tokyo, Japan) with glue, immediately frozen with liquid nitrogen. The samples were transferred to a preparation chamber under vacuum for coating. Photographs of the sample surface were taken using its carrying camera.

### 4.3. Cuticular Wax Analysis

For the wax composition studies, cabbage rosette leaf segments were collected at the harvesting stage and immersed in liquid nitrogen for storage. For wax extraction, 200–400 mg of freeze-dried samples were dipped into 15 mL n-hexane for 30 s at 60 °C and dried under a stream of nitrogen. By adding 100 μL n-hexane, 100 μL N,O-bis (trimethylsilyl) fluoroacetamide (BSTFA), and 2 μL n-tetracosane (20 mg/mL) as an internal standard, samples were shocked for 1 min and subsequently incubated for 30 min at 90 °C. These derivatized samples were then analyzed with an Agilent 7890B-5977B GC-MS (Agilent, Santa Clara, CA, USA).

### 4.4. Fine Mapping of NWGL

Genomic DNA was isolated from young seedlings with normal phenotype. The *NWGL* gene was first mapped to the end of chromosome C08 using 46 F_2_ normal individuals selected from G306×*nwgl* or G274×*nwgl*, respectively. A total of 706 recessive normal individuals selected from the F_2_ population G274×*nwgl* were used for fine mapping. To identify candidate genes, the corresponding DNA fragments were amplified from both *nwgl* and wild-type plants and sequenced. The primer sequences are listed in Table S14.

### 4.5. RNA Isolation, Library Construction and Illumina Sequencing

Total RNA was extracted from collected leaves of *nwgl* and WT plants at harvesting stage using RNAprep Pure Plant kit (Polysaccarides&Polyphenolics-rich) (Tiangen, Beijing, China) according to the manufacturer’s instructions. RNA yield and purity were measured using a NanoDrop 2000 spectrophotometer (Thermo Fisher Scientific, Wilmington, DE, USA). RNA integrity was assessed with an Agilent 2100 Bioanalyser (Agilent, Santa Clara, CA, USA). Six cDNA libraries (three biological replicates per genotype) were constructed using NEBNextUltra^TM^ Directional RNA library Prep kit (NEB, Ipswich, MA, USA) for Illumina (Illumina, San Diego, CA, USA). RNA sequencing was performed by IlluminaHiSeq platform by Biomarker Technologies Co. Ltd. (Biomarker, Beijing, China).

### 4.6. Read Mapping and Transcriptome Assembly

Raw data were filtered using inhouse perl scripts available with Biomarker Technologies Co. Ltd. (Biomarker, Beijing, China). In total, 163.35 Gb clean data were generated from six libraries (on average, more than 26.48 Gb clean data for each sample). Clean reads were aligned to the cabbage genome (www.ocri-genomics.org/bolbase, accessed on: 13 July 2018) using HISAT 2 [47]. The mapped reads were assembled using the StringTie with gff compare program annotation [48]. The raw data were deposited in the GEO database (https://www.ncbi.nlm.nih.gov/geo/, accessed on: 28 April 2019) and the GEO accession number is GSE130405.

### 4.7. Identification of lncRNAs

The unknown transcripts were used for putative lncRNA identification. The lncRNAs candidates were identified followed rigorous criteria: (1) The transcripts length must be ≥200 bp; (2) The transcripts must be with two or more exons; (3) The transcripts must be with FPKM (Fragments Per Kilobase of transcript per Million fragments mapped) value ≥0.1. Then, the predicted lncRNAs were further screened using four computational approaches, including CPC, CNCI, Pfam and CPAT to ensure that the lncRNAs did not have protein-coding ability [49,50,51,52].

### 4.8. Analysis of Differential Expression of Genes and lncRNAs

Genes and lncRNAs expression level was estimated by calculating FPKM values using StringTie (v1.3.1) [48]. Differentially expressed genes and lncRNAs were identified using the DESeq R package (v1.10.1) with ∣log_2_(fold change)∣≥1 and a false discovery rate (FDR) <0.05. The FPKM values of genes and lncRNAs have been deposited in the GEO database (https://www.ncbi.nlm.nih.gov/geo/, accessed on: 28 April 2019) and the GEO accession number is GSE130405.

### 4.9. Target Gene Prediction of lncRNAs

The neighboring genes within 100 Kb upstream and downstream of lncRNAs on the genome were considered as potential *cis-*regulated target genes. The *trans-*regulated target genes were searched using LncTar software based on mRNA sequence complementary and RNA duplex energy prediction [53].

### 4.10. Functional Enrichment Analysis

For all the differentially expressed genes (DEGs) and *cis-*regulated target genes of differentially expressed lncRNAs, Gene Ontology (GO) was analyzed using topGO R packages and Kyoto Encyclopedia of Genes and Genomes (KEGG) analysis was carried out using the KOBAS software [54,55]. Significant enriched Go terms and KEGG pathways were identified with *p* value < 0.05.

### 4.11. Co-expression Analysis

We calculated the Spearman correlation coefficients between the expression levels of differentially expressed lncRNAs and their target genes to analyze their co-expression.

### 4.12. McrBC-, HpaⅡ, MspⅠ-PCR

*Mcr*BC is an endonuclease which cleaves DNA containing methylcytosine and doesnot act upon unmethylated DNA. However, *Mcr*BC cannot recognize *Hpa*Ⅱ/*Msp*Ⅰ endonuclease sites (CCGG) in which the internal cytosine is methylated. For *Mcr*BC, *Hpa*Ⅱ, *Msp*Ⅰ-PCR analysis of *Bol018504*, 1000 ng of genomic DNA was digested using 20 units of the three endonucleases, respectively. The volume of enzyme reaction mixes was 50 μL. The reaction time was 0, 0.5, 3, and 8 h for *Mcr*BC digestion, but 0, and 3 h for *Hpa*Ⅱ and *Msp*Ⅰ digestion. The digested DNAs were used for PCR amplification, and the products were isolated by agarose gel electrophoresis. 

## Figures and Tables

**Table 1:** Cuticular wax composition of leaf blades from wild-type and *nwgl* plants.

Components	Wild-type (μg/g)	*nwgl* (μg/g)
Fatty acid	0.59 ± 0.04	0.32 ± 0.14 *
Aldehyde	12.93 ± 4.33	27.33 ± 13.15
Alkanes	334.36 ± 110.66	5.52 ± 2.97 *
1-Alcohols	35.19 ± 2.74	20.23 ± 12.52
2-Alcohol	14.20 ± 2.49	-
Ketone	174.06 ± 43.09	-
Total	571.33 ± 152.22	53.40 ± 28.31 **

Data are mean ± SD (*n* = 3). * *p* < 0.05, ** *p* < 0.01; Student’s *t*-test.

## Supplementary Materials

Supplementary materials can be found at https://www.mdpi.com/1422-0067/20/11/2820/s1.
