# Supplementary material for: Genome-Wide Analysis of Coding and Long Non-Coding RNAs Involved in Cuticular Wax Biosynthesis in Cabbage (Brassica oleracea L. var. capitata)
Source: Int J Mol Sci. 2019 Jun 10;20(11):2820. doi: 10.3390/ijms20112820 (PMC6600401; doi:10.3390/ijms20112820)
Supplement: Supplementary file 1 [file ijms-20-02820-s001.zip › ijms-505007 supplementary/Supplementary Files/Figure S1. Investigation of methylation level of the Bol018504 gene in nwgl vs. wild-type (WT) plant..pdf]

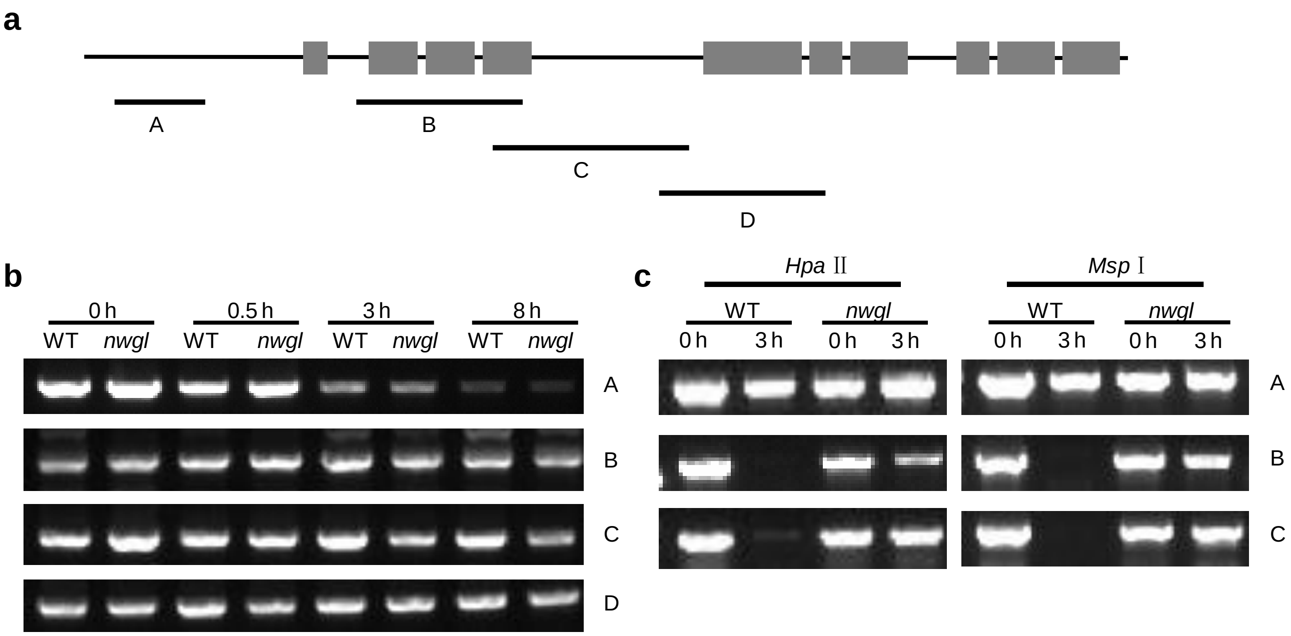

Figure S1. Investigation of methylation level of the *Bol018504* gene in *nwgl* vs. wild-type (WT) plant. (a) Schematic drawing of *Bol018504*. Exons are exhibited as gray boxes, introns and intergenic sequences are exhibited as black lines. Different fragments for the *Mcr*BC- (A to D), *Hpa* II, *Msp* I -PCR (A to C) are shown. (b) *Mcr*BC-PCR analysis of DNA methylation of *Bol018504*. (c) *Hpa* II, *Msp* I -PCR analysis of DNA methylation of *Bol018504*.
