# Supplementary material for: Genome-Wide Analysis of Coding and Long Non-Coding RNAs Involved in Cuticular Wax Biosynthesis in Cabbage (Brassica oleracea L. var. capitata)
Source: Int J Mol Sci. 2019 Jun 10;20(11):2820. doi: 10.3390/ijms20112820 (PMC6600401; doi:10.3390/ijms20112820)
Supplement: Supplementary file 1 [file ijms-20-02820-s001.zip › ijms-505007 supplementary/Supplementary Files/Table S1. The gene information in fine mapping region..pdf]

Table S1. The gene information in fine mapping region.

Gene	Genome version	Physical position	Direction	Gene function annotation
Bol018493	Bolbase V1.0	41430496-41443238	forward	CCR4-Not complex component, Not 1
Bol018494	Bolbase V1.0	41449514-41449945	reverse	There are no functional annotations for this locus
Bol018495	Bolbase V1.0	41450955-41451599	reverse	There are no functional annotations for this locus
Bol018496	Bolbase V1.0	41452950-41453576	reverse	There are no functional annotations for this locus
Bol018497	Bolbase V1.0	41454192-41456273	forward	Replication factor A1
Bol018498	Bolbase V1.0	41462690-41463310	reverse	F-box domain
Bol018499	Bolbase V1.0	41465015-41467031	reverse	COP9 signalosome complex subunit 7
Bol018500	Bolbase V1.0	41467343-41469492	forward	[Phosphatase 2A protein]-leucine-carboxy methyltransferase / Leucine carboxy methyltransferase-1
Bol018501	Bolbase V1.0	41471028-41473980	reverse	Mago nashi protein, Ras-related protein Rab-1A, ADP-ribosylation factor family, GTPase Rab1/YPT1, small G protein superfamily, and related GTP-binding proteins
Bol018502	Bolbase V1.0	41474208-41476458	forward	Dolichyl-P-Man:Man(7)GlcNAc(2)-PP-dolichol alpha-1,6-mannosyltransferase / Dolichyl-PMan:Man(7) GlcNAc (2)-PP-dolichyl alpha-6-mannosyltransferase
Bol018503	Bolbase V1.0	41478952-41479516	forward	Arabidopsis proteins of ECERIFERUM1-LIKE1, fatty acid hydroxylase superfamily
Bol018504	Bolbase V1.0	41482385-41485735	forward	Aldehyde decarbonylase, fatty acid hydroxylase superfamily, wax2 C-terminal domain
Bol018505	Bolbase V1.0	41496061-41496579	reverse	There are no functional annotations for this locus
Bol018506	Bolbase V1.0	41504295-41505063	reverse	Copper transport protein ATOX1-related, ATFP6-LIKE protein-related
Bol018507	Bolbase V1.0	41507930-41514273	reverse	Glutathione peroxidase 4-related, reactive oxygen species degradation
Bol018508	Bolbase V1.0	41507979-41508521	forward	There are no functional annotations for this locus
