# Supplementary material for: Genome-Wide Analysis of Coding and Long Non-Coding RNAs Involved in Cuticular Wax Biosynthesis in Cabbage (Brassica oleracea L. var. capitata)
Source: Int J Mol Sci. 2019 Jun 10;20(11):2820. doi: 10.3390/ijms20112820 (PMC6600401; doi:10.3390/ijms20112820)
Supplement: Supplementary file 1 [file ijms-20-02820-s001.zip › ijms-505007 supplementary/Supplementary Files/Table S10. The trans-regulated target genes of differentially expressed lncRNAs between nwgl and wild-type samples..pdf]

Table S10. The *trans*-regulated target genes of differentially expressed lncRNAs between *nwgl* and wild-type samples.

LncRNA ID	Target genes
MSTRG.84276.1	Bol016968;Bol019540
MSTRG.81531.2	Bol036106
MSTRG.84276.6	Bol016968;Bol019540
MSTRG.52331.1	Bol030628
MSTRG.67492.6	Bol039654;Bol045633
MSTRG.37172.1	Bol035775
MSTRG.20145.1	Bol028016;Bol030273
MSTRG.81713.17	Bol027229
MSTRG.82085.5	Bol042484;Bol027991
MSTRG.63944.1	Bol045154;Bol028371;Bol012694;Bol037706;Bol022908
MSTRG.79055.1	Bol036183;Bol016429;Bol022388;Bol045760;Bol037933;Bol041483
MSTRG.24730.2	Bol032339
MSTRG.82076.6	Bol030273;Bol028016
MSTRG.16553.2	Bol027007;Bol006860;Bol043199;Bol022720;Bol017327;Bol013370;Bol010855;Bol040314;Bol042465;Bol026701;Bol022973;Bol010743;<br>Bol034905;Bol020177;Bol036577
MSTRG.84152.1	Bol036035
MSTRG.15679.1	Bol019375;Bol011136
MSTRG.49742.1	Bol041824;Bol018885;Bol022243;Bol014339;Bol027254;Bol041538;Bol028550;Bol043220;Bol004973;Bol013117;Bol007209;Bol044473;<br>Bol033512;Bol038366
MSTRG.81713.9	Bol033640;Bol027229
MSTRG.81713.24	Bol033640
MSTRG.82076.5	Bol030273;Bol028016
MSTRG.3278.2	Bol025255;Bol031406;Bol025100;Bol044920;Bol036030;Bol016415;Bol007151;Bol022010;Bol032139;Bol016968;Bol038010;Bol009303;<br>Bol017725;Bol035134;Bol029007

MSTRG.83634.13	Bol005793
MSTRG.34912.2	Bol043329;Bol006117;Bol045760;Bol026203;Bol021609;Bol021538;Bol010389
MSTRG.6596.2	Bol024038
MSTRG.82076.4	Bol030273;Bol028016
MSTRG.15660.1	Bol042751;Bol017172;Bol004692;Bol024014;Bol016545;Bol017320;Bol043676;Bol035914
MSTRG.6244.5	Bol030155
MSTRG.71533.1	Bol019759
MSTRG.21778.6	Bol036667
MSTRG.83634.10	Bol005793
MSTRG.63786.1	Bol040358;Bol027347
MSTRG.14102.3	Bol020846
MSTRG.81713.23	Bol027229
MSTRG.6244.7	Bol030155
MSTRG.81713.18	Bol027229
