# Supplementary material for: Genome-Wide Analysis of Coding and Long Non-Coding RNAs Involved in Cuticular Wax Biosynthesis in Cabbage (Brassica oleracea L. var. capitata)
Source: Int J Mol Sci. 2019 Jun 10;20(11):2820. doi: 10.3390/ijms20112820 (PMC6600401; doi:10.3390/ijms20112820)
Supplement: Supplementary file 1 [file ijms-20-02820-s001.zip › ijms-505007 supplementary/Supplementary Files/Table S11. The significant GO terms for cis-regulated target genes of differentially expressed lncRNAs between nwgl and wild-type samples..pdf]

Table S11. The significant GO terms for *cis*-regulatory target genes of differentially expressed lncRNAs between *nwgl* and wild-type samples.

GO ID	GO Term	Go Ontology	Number of Go annotated genes in cluster	Total number of GO annotated genes in cabbage whole genome	*P* value
GO:1901362	organic cyclic compound biosynthetic process	Biological process	19	6061	0.00056
GO:0019438	aromatic compound biosynthetic process	Biological process	17	5669	0.00102
GO:0009909	regulation of flower development	Biological process	2	639	0.00164
GO:0044267	cellular protein metabolic process	Biological process	11	5798	0.0021
GO:0010162	seed dormancy process	Biological process	1	315	0.0023
GO:0045893	positive regulation of transcription, DNA-templated	Biological process	2	778	0.00306
GO:0018130	heterocycle biosynthetic process	Biological process	15	5277	0.00389
GO:1903047	mitotic cell cycle process	Biological process	1	498	0.00449
GO:0048731	system development	Biological process	13	4885	0.00464
GO:0009653	anatomical structure morphogenesis	Biological process	4	3136	0.00478
GO:0048645	organ formation	Biological process	1	398	0.00485
GO:0044271	cellular nitrogen compound biosynthetic process	Biological process	15	5209	0.005
GO:0016569	covalent chromatin modification	Biological process	2	746	0.00581
GO:0006342	chromatin silencing	Biological process	1	444	0.0059
GO:0042537	benzene-containing compound metabolic process	Biological process	2	520	0.00614
GO:0034470	ncRNA processing	Biological process	1	520	0.00628
GO:0010228	vegetative to reproductive phase transition of meristem	Biological process	1	808	0.00651
GO:0098662	inorganic cation transmembrane transport	Biological process	1	325	0.00701
GO:0009699	phenylpropanoid biosynthetic process	Biological process	1	292	0.0079
GO:0006479	protein methylation	Biological process	1	538	0.00816
GO:0051171	regulation of nitrogen compound metabolic process	Biological process	13	3973	0.00833
GO:0080090	regulation of primary metabolic process	Biological process	14	4611	0.00834

GO:2000112	regulation of cellular macromolecule biosynthetic process	Biological process	14	3888	0.00869
GO:0030001	metal ion transport	Biological process	1	1082	0.00893
GO:0043086	negative regulation of catalytic activity	Biological process	2	280	0.00988
GO:0044281	small molecule metabolic process	Biological process	11	5596	0.01002
GO:0019219	regulation of nucleobase-containing compound metabolic process	Biological process	13	3915	0.01047
GO:1902410	mitotic cytokinetic process	Biological process	1	362	0.01063
GO:0000911	cytokinesis by cell plate formation	Biological process	1	362	0.01063
GO:0000281	mitotic cytokinesis	Biological process	1	362	0.01063
GO:0048513	organ development	Biological process	10	3476	0.01126
GO:0034645	cellular macromolecule biosynthetic process	Biological process	16	6438	0.01146
GO:0061640	cytoskeleton-dependent cytokinesis	Biological process	1	699	0.01156
GO:0006396	RNA processing	Biological process	3	1403	0.01185
GO:1900673	olefin metabolic process	Biological process	1	203	0.01229
GO:1900674	olefin biosynthetic process	Biological process	1	203	0.01229
GO:0043450	alkene biosynthetic process	Biological process	1	203	0.01229
GO:0009692	ethylene metabolic process	Biological process	1	203	0.01229
GO:0009693	ethylene biosynthetic process	Biological process	1	203	0.01229
GO:0043449	cellular alkene metabolic process	Biological process	1	203	0.01229
GO:0000910	cytokinesis	Biological process	1	739	0.01314
GO:0005975	carbohydrate metabolic process	Biological process	8	3346	0.01325
GO:0009059	macromolecule biosynthetic process	Biological process	16	6602	0.01351
GO:0048507	meristem development	Biological process	3	956	0.01368
GO:0006928	cellular component movement	Biological process	1	219	0.01378
GO:0032506	cytokinetic process	Biological process	1	370	0.01408
GO:0015740	C4-dicarboxylate transport	Biological process	1	24	0.01479

GO:0015743	malate transport	Biological process	1	24	0.01479
GO:0009697	salicylic acid biosynthetic process	Biological process	2	420	0.01492
GO:1901659	glycosyl compound biosynthetic process	Biological process	1	493	0.01498
GO:0016571	histone methylation	Biological process	1	523	0.01516
GO:0034654	nucleobase-containing compound biosynthetic process	Biological process	13	4621	0.01601
GO:0016052	carbohydrate catabolic process	Biological process	1	1123	0.01616
GO:0009056	catabolic process	Biological process	6	4089	0.01695
GO:0010467	gene expression	Biological process	15	6016	0.01734
GO:0010038	response to metal ion	Biological process	3	1295	0.0185
GO:1901657	glycosyl compound metabolic process	Biological process	1	1300	0.01989
GO:0009410	response to xenobiotic stimulus	Biological process	1	137	0.02078
GO:0009698	phenylpropanoid metabolic process	Biological process	1	402	0.0209
GO:0032774	RNA biosynthetic process	Biological process	13	3984	0.02155
GO:0090351	seedling development	Biological process	2	480	0.02252
GO:0048827	phyllome development	Biological process	5	1511	0.0236
GO:0006355	regulation of transcription, DNA-templated	Biological process	13	3636	0.02435
GO:2001141	regulation of RNA biosynthetic process	Biological process	13	3636	0.02435
GO:0006351	transcription, DNA-templated	Biological process	13	3981	0.02469
GO:0046189	phenol-containing compound biosynthetic process	Biological process	2	438	0.02521
GO:0010039	response to iron ion	Biological process	1	92	0.02644
GO:0009887	organ morphogenesis	Biological process	2	925	0.02858
GO:0018958	phenol-containing compound metabolic process	Biological process	2	464	0.02915
GO:0010468	regulation of gene expression	Biological process	14	4107	0.02958
GO:0048563	post-embryonic organ morphogenesis	Biological process	1	329	0.02973
GO:0048444	floral organ morphogenesis	Biological process	1	329	0.02973
GO:0042343	indole glucosinolate metabolic process	Biological process	1	44	0.0302

GO:0009696	salicylic acid metabolic process	Biological process	2	442	0.03147
GO:0016070	RNA metabolic process	Biological process	15	5603	0.03151
GO:0032787	monocarboxylic acid metabolic process	Biological process	8	2547	0.03188
GO:0044723	single-organism carbohydrate metabolic process	Biological process	5	2679	0.03227
GO:0016144	S-glycoside biosynthetic process	Biological process	1	318	0.03229
GO:0019761	glucosinolate biosynthetic process	Biological process	1	318	0.03229
GO:0019758	glycosinolate biosynthetic process	Biological process	1	318	0.03229
GO:0016570	histone modification	Biological process	1	643	0.03299
GO:0007389	pattern specification process	Biological process	3	659	0.03307
GO:0051252	regulation of RNA metabolic process	Biological process	13	3648	0.03451
GO:1901135	carbohydrate derivative metabolic process	Biological process	3	2570	0.03543
GO:0010218	response to far red light	Biological process	2	181	0.03646
GO:0048829	root cap development	Biological process	1	28	0.03909
GO:0048509	regulation of meristem development	Biological process	1	420	0.03918
GO:0010073	meristem maintenance	Biological process	2	548	0.03943
GO:0048367	shoot system development	Biological process	6	2423	0.04051
GO:0048193	Golgi vesicle transport	Biological process	1	670	0.04058
GO:0030048	actin filament-based movement	Biological process	1	128	0.0409
GO:0098656	anion transmembrane transport	Biological process	1	139	0.04091
GO:0008380	RNA splicing	Biological process	1	471	0.04222
GO:0046486	glycerolipid metabolic process	Biological process	1	447	0.04254
GO:0034220	ion transmembrane transport	Biological process	3	529	0.04281
GO:0009886	post-embryonic morphogenesis	Biological process	4	807	0.04323
GO:1901616	organic hydroxy compound catabolic process	Biological process	1	74	0.04434
GO:0043543	protein acylation	Biological process	1	307	0.0464
GO:0044085	cellular component biogenesis	Biological process	2	2574	0.04662

GO:0006364	rRNA processing	Biological process	1	426	0.04705
GO:0009651	response to salt stress	Biological process	5	1625	0.04767
GO:1901698	response to nitrogen compound	Biological process	6	1566	0.04799
GO:0009723	response to ethylene	Biological process	3	696	0.04819
GO:0005634	nucleus	Cellular Component	21	11418	0.0056
GO:0005856	cytoskeleton	Cellular Component	1	412	0.0294
GO:0005515	protein binding	Molecular Function	16	4532	1.70E-05
GO:0003700	sequence-specific DNA binding transcription factor activity	Molecular Function	9	2365	0.00337
GO:0015197	peptide transporter activity	Molecular Function	1	44	0.00341
GO:0003677	DNA binding	Molecular Function	9	2695	0.00672
GO:0004175	endopeptidase activity	Molecular Function	1	370	0.01174
GO:0015198	oligopeptide transporter activity	Molecular Function	1	34	0.01427
GO:0004252	serine-type endopeptidase activity	Molecular Function	1	129	0.01462
GO:0020037	heme binding	Molecular Function	1	409	0.01834
GO:0043565	sequence-specific DNA binding	Molecular Function	2	564	0.02594
GO:0004712	protein serine/threonine/tyrosine kinase activity	Molecular Function	1	82	0.02795
GO:0003779	actin binding	Molecular Function	2	129	0.02915
GO:0008092	cytoskeletal protein binding	Molecular Function	2	292	0.02976
GO:0005381	iron ion transmembrane transporter activity	Molecular Function	1	21	0.04625
GO:0042802	identical protein binding	Molecular Function	2	380	0.04746
GO:0008324	cation transmembrane transporter activity	Molecular Function	2	737	0.04855
