# Supplementary material for: Genome-Wide Analysis of Coding and Long Non-Coding RNAs Involved in Cuticular Wax Biosynthesis in Cabbage (Brassica oleracea L. var. capitata)
Source: Int J Mol Sci. 2019 Jun 10;20(11):2820. doi: 10.3390/ijms20112820 (PMC6600401; doi:10.3390/ijms20112820)
Supplement: Supplementary file 1 [file ijms-20-02820-s001.zip › ijms-505007 supplementary/Supplementary Files/Table S12. Functional enrichment analysis for cis-regulated target genes of differentially expressed lncRNAs bwtween nwgl and wild-type samples based on KEGG metabolic pathways..pdf]

Table S12. Functional enrichment analysis for *cis*-regulated target genes of differentially expressed lncRNAs bwtween *nwgl* and wild-type samples based on KEGG metabolic pathways.

KEGG ID	KEGG pathway	Number of KEGG annotated genes in cluster	Total number of KEGG annotated genes in cabbage whole genome	*P* value	gene
ko00030	Pentose phosphate pathway	9	77	0.000145153	Bol014834;Bol014835;Bol014852;Bol022230;Bol025079;Bol025168;Bol027442;Bol032220;Bol043460
ko04144	Endocytosis	14	198	0.000552854	Bol008666;Bol009378;Bol012097;Bol022060;Bol025141;Bol025647;Bol029613;Bol038852;Bol039247;Bol043548;Bol043549;Bol043675;Bol043759;Bol045856
ko03010	Ribosome	22	509	0.011025865	Bol009313;Bol009384;Bol014842;Bol016098;Bol017042;Bol017267;Bol020097;Bol023249;Bol025146;Bol025158;Bol025650;Bol026085;Bol028739;Bol029383;Bol030275;Bol032335;Bol032858;Bol036551;Bol042926;Bol042927;Bol043135;Bol044778
ko00010	Glycolysis / Gluconeogenesis	10	172	0.013070961	Bol008679;Bol014852;Bol016436;Bol021928;Bol025079;Bol025168;Bol030263;Bol038918;Bol043779;Bol044773
ko01220	Degradation of aromatic compounds	2	9	0.021040719	Bol016436;Bol044773
ko00190	Oxidative phosphorylation	12	246	0.024578621	Bol009394;Bol019295;Bol025064;Bol025136;Bol025645;Bol032215;Bol032937;Bol043546;Bol043739;Bol043770;Bol043771;Bol043784
