# Supplementary material for: Genome-Wide Analysis of Coding and Long Non-Coding RNAs Involved in Cuticular Wax Biosynthesis in Cabbage (Brassica oleracea L. var. capitata)
Source: Int J Mol Sci. 2019 Jun 10;20(11):2820. doi: 10.3390/ijms20112820 (PMC6600401; doi:10.3390/ijms20112820)
Supplement: Supplementary file 1 [file ijms-20-02820-s001.zip › ijms-505007 supplementary/Supplementary Files/Table S14. List of primers used in this study..pdf]

Table S14. List of primers used in this study.

Primer name	Sequence (5'-3')	Used for
at040_F	AAGAGTGGATCGGTGTGGAGTG	Mapping
at040_R	TTGAGGAAGTGGCTAAGCATGA	Mapping
ct011_F	AAATTGAAGAAACGTTAAAGCAAAA	Mapping
ct011_R	TAATCGTAGAGGGAAAGAGAGAGAG	Mapping
at006_F	CGTTGCCGGTGATCTCCTTCGTA	Mapping
at006_R	GCATCGTTTTGCTCCCCTTCTAT	Mapping
at008_F	TAGCCTTCTTTGAGACTTCATACCA	Mapping
at008_R	GTACACCTTCTTAGTCCAGTCCATC	Mapping
ID25_F	CTCTAGAGGAATCCAAGCGAAGG	Mapping
ID25_R	AGAGAGAGAGAACTGAAGGAAGGA	Mapping
at013_F	ATCAATGCCAAACGGAGAGGACG	Mapping
at013_R	ATCAATGACGCAAATGAGACGAG	Mapping
at004_F	TTTTTGTCGTCGCTCTGGCACCA	Mapping
at004_R	GCAACCGCTTTCAACACTCGTAC	Mapping
