# Supplementary material for: Genome-Wide Analysis of Coding and Long Non-Coding RNAs Involved in Cuticular Wax Biosynthesis in Cabbage (Brassica oleracea L. var. capitata)
Source: Int J Mol Sci. 2019 Jun 10;20(11):2820. doi: 10.3390/ijms20112820 (PMC6600401; doi:10.3390/ijms20112820)
Supplement: Supplementary file 1 [file ijms-20-02820-s001.zip › ijms-505007 supplementary/Supplementary Files/Table S3. GO enrichment analysis for down-regulated genes in plants exhibiting nwgl phenotype..pdf]

Table S3. GO enrichment analysis for down-regulated genes in plants exhibiting *nwgl* phenotype.

GO ID	GO Term	Go Ontology	Number of Go annotated genes in cluster	Total number of GO annotated genes in cabbage whole genome	*P* value
GO:0010025	wax biosynthetic process	Biological Process	2	42	9.30E-05
GO:0006265	DNA topological change	Biological Process	2	18	0.00014
GO:0009887	animal organ morphogenesis	Biological Process	19	925	0.00054
GO:0070918	production of small RNA involved in gene silencing by RNA	Biological Process	1	233	0.00062
GO:0010267	production of ta-siRNAs involved in RNA interference	Biological Process	1	172	0.00064
GO:0010073	meristem maintenance	Biological Process	22	548	0.00093
GO:0009793	embryo development ending in seed dormancy	Biological Process	6	962	0.00124
GO:0016246	RNA interference	Biological Process	3	302	0.00129
GO:0006355	regulation of transcription, DNA-templated	Biological Process	50	3636	0.00141
GO:0048509	regulation of meristem development	Biological Process	22	420	0.00217
GO:0010162	seed dormancy process	Biological Process	1	315	0.00233
GO:0009909	regulation of flower development	Biological Process	9	639	0.00283
GO:0048729	tissue morphogenesis	Biological Process	1	10	0.00292
GO:0006812	cation transport	Biological Process	12	1587	0.00312
GO:0035196	production of miRNAs involved in gene silencing by miRNA	Biological Process	1	212	0.00326
GO:0030154	cell differentiation	Biological Process	20	2068	0.00338
GO:1901362	organic cyclic compound biosynthetic process	Biological Process	72	6061	0.00352
GO:0010192	mucilage biosynthetic process	Biological Process	1	17	0.00493
GO:0048451	petal formation	Biological Process	3	100	0.00498
GO:0048453	sepal formation	Biological Process	3	100	0.00498
GO:0052542	defense response by callose deposition	Biological Process	1	141	0.00517
GO:0048653	anther development	Biological Process	5	159	0.00529

GO:0010027	thylakoid membrane organization	Biological Process	1	285	0.00534
GO:0016052	carbohydrate catabolic process	Biological Process	9	1123	0.00613
GO:0044267	cellular protein metabolic process	Biological Process	40	5798	0.00615
GO:0010214	seed coat development	Biological Process	3	42	0.00636
GO:0009610	response to symbiotic fungus	Biological Process	1	85	0.00642
GO:0035266	meristem growth	Biological Process	21	358	0.00643
GO:0034470	ncRNA processing	Biological Process	2	520	0.00645
GO:0006306	DNA methylation	Biological Process	6	298	0.00662
GO:0009250	glucan biosynthetic process	Biological Process	10	507	0.00683
GO:0045893	positive regulation of transcription, DNA-templated	Biological Process	9	778	0.00702
GO:0010182	sugar mediated signaling pathway	Biological Process	1	235	0.00723
GO:0000911	cytokinesis by cell plate formation	Biological Process	4	362	0.00783
GO:1901659	glycosyl compound biosynthetic process	Biological Process	15	493	0.00883
GO:0009653	anatomical structure morphogenesis	Biological Process	37	3136	0.00906
GO:0009699	phenylpropanoid biosynthetic process	Biological Process	1	292	0.00957
GO:0044281	small molecule metabolic process	Biological Process	50	5596	0.00961
GO:0042127	regulation of cell proliferation	Biological Process	3	212	0.00992
GO:0030001	metal ion transport	Biological Process	7	1083	0.01045
GO:0045814	negative regulation of gene expression, epigenetic	Biological Process	6	447	0.01056
GO:0040034	regulation of development, heterochronic	Biological Process	1	69	0.01061
GO:0043086	negative regulation of catalytic activity	Biological Process	7	280	0.01061
GO:0006342	chromatin silencing	Biological Process	6	444	0.01124
GO:0048829	root cap development	Biological Process	2	28	0.01188
GO:0015740	C4-dicarboxylate transport	Biological Process	1	24	0.01209
GO:0015743	malate transport	Biological Process	1	24	0.01209
GO:0010228	vegetative to reproductive phase transition of meristem	Biological Process	1	808	0.01266

GO:0006007	glucose catabolic process	Biological Process	4	509	0.01268
GO:0046365	monosaccharide catabolic process	Biological Process	4	509	0.01268
GO:0019320	hexose catabolic process	Biological Process	4	509	0.01268
GO:0009116	nucleoside metabolic process	Biological Process	6	896	0.01281
GO:0009567	double fertilization forming a zygote and endosperm	Biological Process	1	50	0.01283
GO:0000278	mitotic cell cycle	Biological Process	8	739	0.01355
GO:0019438	aromatic compound biosynthetic process	Biological Process	60	5669	0.01402
GO:0006325	chromatin organization	Biological Process	11	950	0.01412
GO:0048588	developmental cell growth	Biological Process	8	852	0.01412
GO:0019761	glucosinolate biosynthetic process	Biological Process	13	318	0.01458
GO:0019758	glycosinolate biosynthetic process	Biological Process	13	318	0.01458
GO:0016144	S-glycoside biosynthetic process	Biological Process	13	318	0.01458
GO:0006928	movement of cell or subcellular component	Biological Process	3	219	0.01503
GO:0010075	regulation of meristem growth	Biological Process	21	333	0.01516
GO:0009833	plant-type primary cell wall biogenesis	Biological Process	1	9	0.0155
GO:0015851	nucleobase transport	Biological Process	1	117	0.01647
GO:0016568	chromatin organization	Biological Process	10	798	0.01648
GO:0002009	morphogenesis of an epithelium	Biological Process	1	7	0.01683
GO:0001738	morphogenesis of a polarized epithelium	Biological Process	1	7	0.01683
GO:0034220	ion transmembrane transport	Biological Process	9	529	0.01746
GO:0010016	shoot system morphogenesis	Biological Process	17	847	0.01757
GO:0030422	production of siRNA involved in RNA interference	Biological Process	1	224	0.01795
GO:0051274	beta-glucan biosynthetic process	Biological Process	2	206	0.01813
GO:0071322	cellular response to carbohydrate stimulus	Biological Process	1	249	0.01903
GO:0044724	single-organism carbohydrate catabolic process	Biological Process	5	972	0.01946
GO:0055086	nucleobase-containing small molecule metabolic process	Biological Process	12	1805	0.01964

GO:0051325	interphase	Biological Process	1	125	0.01979
GO:0051329	mitotic interphase	Biological Process	1	125	0.01979
GO:0051319	G2 phase	Biological Process	1	125	0.01979
GO:0022403	cell cycle phase	Biological Process	1	125	0.01979
GO:0044848	biological phase	Biological Process	1	125	0.01979
GO:0000085	mitotic G2 phase	Biological Process	1	125	0.01979
GO:0009933	meristem structural organization	Biological Process	6	484	0.02008
GO:0009808	lignin metabolic process	Biological Process	2	158	0.02019
GO:0018130	heterocycle biosynthetic process	Biological Process	61	5277	0.02042
GO:0016569	covalent chromatin modification	Biological Process	9	746	0.02215
GO:0048366	leaf development	Biological Process	12	827	0.02217
GO:0009110	vitamin biosynthetic process	Biological Process	1	90	0.02246
GO:0048359	mucilage metabolic process involved in seed coat development	Biological Process	1	19	0.02278
GO:0072521	purine-containing compound metabolic process	Biological Process	6	952	0.02288
GO:0042364	water-soluble vitamin biosynthetic process	Biological Process	1	81	0.02298
GO:0046128	purine ribonucleoside metabolic process	Biological Process	6	835	0.02341
GO:0006863	purine nucleobase transport	Biological Process	1	115	0.02393
GO:0009119	ribonucleoside metabolic process	Biological Process	6	868	0.02434
GO:0042278	purine nucleoside metabolic process	Biological Process	6	839	0.02457
GO:0045962	positive regulation of development, heterochronic	Biological Process	1	12	0.02515
GO:0007264	small GTPase mediated signal transduction	Biological Process	1	232	0.02519
GO:0048646	anatomical structure formation involved in morphogenesis	Biological Process	11	584	0.02583
GO:0044550	secondary metabolite biosynthetic process	Biological Process	14	617	0.02661
GO:0044092	negative regulation of molecular function	Biological Process	7	304	0.02661
GO:0007017	microtubule-based process	Biological Process	7	547	0.02663
GO:0051273	beta-glucan metabolic process	Biological Process	4	315	0.02715

GO:0043900	regulation of multi-organism process	Biological Process	1	290	0.02732
GO:0008213	protein alkylation	Biological Process	6	538	0.02735
GO:0006479	protein methylation	Biological Process	6	538	0.02735
GO:0010067	procambium histogenesis	Biological Process	2	12	0.02744
GO:0048468	cell development	Biological Process	14	1362	0.02766
GO:0006006	glucose metabolic process	Biological Process	4	868	0.02783
GO:0006163	purine nucleotide metabolic process	Biological Process	5	899	0.02827
GO:0048532	anatomical structure arrangement	Biological Process	6	492	0.02842
GO:0009934	regulation of meristem structural organization	Biological Process	1	30	0.02854
GO:0010038	response to metal ion	Biological Process	9	1295	0.0291
GO:0009117	nucleotide metabolic process	Biological Process	11	1724	0.02916
GO:0048645	animal organ formation	Biological Process	8	398	0.02979
GO:0044249	cellular biosynthetic process	Biological Process	103	10046	0.03
GO:0019693	ribose phosphate metabolic process	Biological Process	6	1070	0.03006
GO:0009259	ribonucleotide metabolic process	Biological Process	6	1070	0.03006
GO:0050790	regulation of catalytic activity	Biological Process	11	678	0.03158
GO:0010101	post-embryonic root morphogenesis	Biological Process	1	100	0.03186
GO:0010102	lateral root morphogenesis	Biological Process	1	100	0.03186
GO:0006753	nucleoside phosphate metabolic process	Biological Process	11	1731	0.03205
GO:0030048	actin filament-based movement	Biological Process	2	128	0.03223
GO:0009692	ethylene metabolic process	Biological Process	1	203	0.03247
GO:0009693	ethylene biosynthetic process	Biological Process	1	203	0.03247
GO:0043450	alkene biosynthetic process	Biological Process	1	203	0.03247
GO:0043449	cellular alkene metabolic process	Biological Process	1	203	0.03247
GO:1900673	olefin metabolic process	Biological Process	1	203	0.03247
GO:1900674	olefin biosynthetic process	Biological Process	1	203	0.03247

GO:0080147	root hair cell development	Biological Process	5	434	0.03282
GO:0009566	fertilization	Biological Process	1	57	0.03357
GO:0006346	methylation-dependent chromatin silencing	Biological Process	3	223	0.03387
GO:0006767	water-soluble vitamin metabolic process	Biological Process	1	82	0.03403
GO:0008356	asymmetric cell division	Biological Process	4	42	0.03427
GO:0007389	pattern specification process	Biological Process	11	659	0.03428
GO:0006396	RNA processing	Biological Process	4	1403	0.03434
GO:0098662	inorganic cation transmembrane transport	Biological Process	5	325	0.03468
GO:0048465	corolla development	Biological Process	4	161	0.03547
GO:0048441	petal development	Biological Process	4	161	0.03547
GO:0009965	leaf morphogenesis	Biological Process	9	402	0.03562
GO:0009150	purine ribonucleotide metabolic process	Biological Process	5	834	0.03608
GO:0098655	cation transmembrane transport	Biological Process	5	334	0.03855
GO:0044723	single-organism carbohydrate metabolic process	Biological Process	24	2679	0.03936
GO:0030244	cellulose biosynthetic process	Biological Process	2	185	0.03974
GO:0005996	monosaccharide metabolic process	Biological Process	5	1021	0.04113
GO:0005982	starch metabolic process	Biological Process	10	384	0.04143
GO:0065009	regulation of molecular function	Biological Process	13	782	0.0422
GO:0009698	phenylpropanoid metabolic process	Biological Process	2	402	0.0423
GO:0044272	sulfur compound biosynthetic process	Biological Process	21	935	0.04349
GO:0019318	hexose metabolic process	Biological Process	4	926	0.04411
GO:1901657	glycosyl compound metabolic process	Biological Process	19	1300	0.04456
GO:0031537	regulation of anthocyanin metabolic process	Biological Process	3	57	0.04464
GO:0009615	response to virus	Biological Process	4	311	0.0451
GO:0009205	purine ribonucleoside triphosphate metabolic process	Biological Process	5	764	0.04554
GO:0009144	purine nucleoside triphosphate metabolic process	Biological Process	5	764	0.04554

GO:0009798	axis specification	Biological Process	4	161	0.04561
GO:0009723	response to ethylene	Biological Process	4	696	0.04589
GO:0006790	sulfur compound metabolic process	Biological Process	27	1216	0.04619
GO:0071370	cellular response to gibberellin stimulus	Biological Process	3	152	0.04672
GO:0032787	monocarboxylic acid metabolic process	Biological Process	11	2547	0.04765
GO:0044712	single-organism catabolic process	Biological Process	17	2895	0.04773
GO:0009809	lignin biosynthetic process	Biological Process	1	113	0.04818
GO:0065001	specification of axis polarity	Biological Process	4	141	0.04828
GO:0016571	histone methylation	Biological Process	6	523	0.04829
GO:0010476	gibberellin mediated signaling pathway	Biological Process	3	150	0.04996
GO:0009504	cell plate	Cellular Component	1	73	0.0007
GO:0005634	nucleus	Cellular Component	101	11418	0.0049
GO:0009295	nucleoid	Cellular Component	1	57	0.0178
GO:0031225	anchored component of membrane	Cellular Component	5	296	0.026
GO:0010330	cellulose synthase complex	Cellular Component	1	3	0.036
GO:0005783	endoplasmic reticulum	Cellular Component	6	1005	0.0449
GO:0009505	plant-type cell wall	Cellular Component	9	510	0.0494
GO:0005515	protein binding	Molecular Function	42	4532	6.20E-05
GO:0003916	DNA topoisomerase activity	Molecular Function	2	18	0.00015
GO:0003700	transcription factor activity, sequence-specific DNA binding	Molecular Function	39	2365	0.00041
GO:0005525	GTP binding	Molecular Function	1	370	0.00117
GO:0010177	2-(2'-methylthio)ethylmalate synthase activity	Molecular Function	2	10	0.00277
GO:0004252	serine-type endopeptidase activity	Molecular Function	7	129	0.00631
GO:0020037	heme binding	Molecular Function	9	409	0.00787
GO:0003677	DNA binding	Molecular Function	31	2695	0.00955
GO:0050897	cobalt ion binding	Molecular Function	2	83	0.01218

GO:0005345	purine nucleobase transmembrane transporter activity	Molecular Function	1	26	0.01242
GO:0003779	actin binding	Molecular Function	3	129	0.01314
GO:0008378	galactosyltransferase activity	Molecular Function	1	54	0.01351
GO:0016765	transferase activity, transferring alkyl or aryl (other than methyl) groups	Molecular Function	1	157	0.01486
GO:0003918	DNA topoisomerase type II (ATP-hydrolyzing) activity	Molecular Function	1	12	0.0172
GO:0061505	DNA topoisomerase II activity	Molecular Function	1	12	0.0172
GO:0003917	DNA topoisomerase type I activity	Molecular Function	1	9	0.01783
GO:0008092	cytoskeletal protein binding	Molecular Function	6	292	0.02405
GO:0008134	transcription factor binding	Molecular Function	1	52	0.03004
GO:0042802	identical protein binding	Molecular Function	8	380	0.03048
GO:0004175	endopeptidase activity	Molecular Function	13	370	0.03145
GO:0043565	sequence-specific DNA binding	Molecular Function	7	564	0.03373
GO:0008762	UDP-N-acetylmuramate dehydrogenase activity	Molecular Function	1	62	0.03615
GO:0004712	protein serine/threonine/tyrosine kinase activity	Molecular Function	1	82	0.03912
GO:0005381	iron ion transmembrane transporter activity	Molecular Function	1	21	0.04009
GO:0004650	polygalacturonase activity	Molecular Function	2	99	0.0426
GO:0016760	cellulose synthase (UDP-forming) activity	Molecular Function	2	30	0.04616
GO:0015205	nucleobase transmembrane transporter activity	Molecular Function	1	31	0.04678
