# Supplementary material for: Genome-Wide Analysis of Coding and Long Non-Coding RNAs Involved in Cuticular Wax Biosynthesis in Cabbage (Brassica oleracea L. var. capitata)
Source: Int J Mol Sci. 2019 Jun 10;20(11):2820. doi: 10.3390/ijms20112820 (PMC6600401; doi:10.3390/ijms20112820)
Supplement: Supplementary file 1 [file ijms-20-02820-s001.zip › ijms-505007 supplementary/Supplementary Files/Table S4. GO enrichment analysis for up-regulated genes in plants exhibiting nwgl phenotype..pdf]

Table S4. GO enrichment analysis for up-regulated genes in plants exhibiting *nwgl* phenotype.

GO ID	GO Term	Go Ontology	Number of Go annotated genes incluster	Total number of GO annotated genes in cabbage whole genome	*P* value
GO:0010200	response to chitin	Biological Process	152	851	2.50E-18
GO:0002679	respiratory burst involved in defense response	Biological Process	64	247	6.70E-13
GO:0050832	defense response to fungus	Biological Process	88	691	6.60E-08
GO:0009697	salicylic acid biosynthetic process	Biological Process	59	420	2.80E-07
GO:0010363	regulation of plant-type hypersensitive response	Biological Process	94	759	4.40E-07
GO:0006612	protein targeting to membrane	Biological Process	95	785	6.40E-07
GO:0000165	MAPK cascade	Biological Process	65	478	7.80E-07
GO:0009862	systemic acquired resistance, salicylic acid mediated signaling pathway	Biological Process	69	502	1.00E-06
GO:0043069	negative regulation of programmed cell death	Biological Process	58	355	1.60E-06
GO:0031348	negative regulation of defense response	Biological Process	76	566	4.60E-06
GO:0051865	protein autoubiquitination	Biological Process	5	26	8.30E-06
GO:0030968	endoplasmic reticulum unfolded protein response	Biological Process	48	365	9.70E-06
GO:0009693	ethylene biosynthetic process	Biological Process	23	203	1.10E-05
GO:0043900	regulation of multi-organism process	Biological Process	32	290	3.20E-05
GO:0009867	jasmonic acid mediated signaling pathway	Biological Process	73	585	4.20E-05
GO:0009738	abscisic acid-activated signaling pathway	Biological Process	58	532	6.30E-05
GO:0006355	regulation of transcription, DNA-templated	Biological Process	143	3636	6.90E-05
GO:0009723	response to ethylene	Biological Process	70	696	0.0001
GO:1900056	negative regulation of leaf senescence	Biological Process	7	12	0.00024
GO:0009595	detection of biotic stimulus	Biological Process	32	216	0.00031
GO:0010310	regulation of hydrogen peroxide metabolic process	Biological Process	44	358	0.00034

GO:0009414	response to water deprivation	Biological Process	65	842	0.00036
GO:0051245	negative regulation of cellular defense response	Biological Process	3	4	0.00065
GO:0015858	nucleoside transport	Biological Process	3	21	0.00089
GO:0009611	response to wounding	Biological Process	67	696	0.00112
GO:0009887	animal organ morphogenesis	Biological Process	9	925	0.00126
GO:0010192	mucilage biosynthetic process	Biological Process	1	17	0.0014
GO:0000278	mitotic cell cycle	Biological Process	9	739	0.00199
GO:0009871	jasmonic acid and ethylene-dependent systemic resistance, ethylene mediated signaling pathway	Biological Process	1	7	0.00214
GO:0035556	intracellular signal transduction	Biological Process	120	1281	0.00254
GO:0009625	response to insect	Biological Process	20	151	0.00287
GO:0006812	cation transport	Biological Process	55	1587	0.00345
GO:0042742	defense response to bacterium	Biological Process	73	860	0.0039
GO:0042538	hyperosmotic salinity response	Biological Process	33	342	0.00399
GO:0015700	arsenite transport	Biological Process	1	16	0.00402
GO:0009864	induced systemic resistance, jasmonic acid mediated signaling pathway	Biological Process	5	21	0.00444
GO:0016246	RNA interference	Biological Process	1	302	0.00464
GO:0052542	defense response by callose deposition	Biological Process	16	141	0.00492
GO:0048316	seed development	Biological Process	13	1231	0.00528
GO:0044003	modification by symbiont of host morphology or physiology	Biological Process	1	199	0.00534
GO:0010508	positive regulation of autophagy	Biological Process	3	7	0.0054
GO:0009790	embryo development	Biological Process	12	1106	0.00546
GO:0035195	gene silencing by miRNA	Biological Process	1	224	0.00571
GO:0010286	heat acclimation	Biological Process	21	156	0.00611
GO:0009610	response to symbiotic fungus	Biological Process	2	85	0.00623
GO:0051707	response to other organism	Biological Process	206	2672	0.00626

GO:0009808	lignin metabolic process	Biological Process	9	158	0.00632
GO:0009410	response to xenobiotic stimulus	Biological Process	6	137	0.0066
GO:0046168	glycerol-3-phosphate catabolic process	Biological Process	1	5	0.00685
GO:0009863	salicylic acid mediated signaling pathway	Biological Process	85	690	0.00722
GO:0051607	defense response to virus	Biological Process	7	254	0.00776
GO:0006952	defense response	Biological Process	236	2882	0.00786
GO:0044550	secondary metabolite biosynthetic process	Biological Process	24	617	0.008
GO:0009893	positive regulation of metabolic process	Biological Process	47	1162	0.0086
GO:0071329	cellular response to sucrose stimulus	Biological Process	2	15	0.00895
GO:0006487	protein N-linked glycosylation	Biological Process	5	189	0.00906
GO:0042537	benzene-containing compound metabolic process	Biological Process	65	520	0.00933
GO:0010876	lipid localization	Biological Process	13	407	0.00941
GO:0009963	positive regulation of flavonoid biosynthetic process	Biological Process	21	234	0.00978
GO:0051592	response to calcium ion	Biological Process	2	26	0.01012
GO:0009699	phenylpropanoid biosynthetic process	Biological Process	15	292	0.01081
GO:0035196	production of miRNAs involved in gene silencing by miRNA	Biological Process	1	212	0.01085
GO:0031325	positive regulation of cellular metabolic process	Biological Process	26	918	0.01091
GO:0045923	positive regulation of fatty acid metabolic process	Biological Process	2	3	0.01103
GO:0045723	positive regulation of fatty acid biosynthetic process	Biological Process	2	3	0.01103
GO:0009743	response to carbohydrate	Biological Process	24	788	0.01106
GO:0010941	regulation of cell death	Biological Process	99	831	0.01115
GO:0010431	seed maturation	Biological Process	3	369	0.01216
GO:0009793	embryo development ending in seed dormancy	Biological Process	10	962	0.01233
GO:0050691	regulation of defense response to virus by host	Biological Process	3	4	0.01238
GO:0010162	seed dormancy process	Biological Process	3	315	0.01254
GO:0022611	dormancy process	Biological Process	3	315	0.01254

GO:0051239	regulation of multicellular organismal process	Biological Process	21	1364	0.0128
GO:0080026	response to indolebutyric acid	Biological Process	1	15	0.01313
GO:0031050	dsRNA fragmentation	Biological Process	1	233	0.01379
GO:0043331	response to dsRNA	Biological Process	1	233	0.01379
GO:0071359	cellular response to dsRNA	Biological Process	1	233	0.01379
GO:0070918	production of small RNA involved in gene silencing by RNA	Biological Process	1	233	0.01379
GO:0071310	cellular response to organic substance	Biological Process	163	2439	0.01415
GO:0016311	dephosphorylation	Biological Process	28	469	0.01453
GO:0015867	ATP transport	Biological Process	1	11	0.01476
GO:0015868	purine ribonucleotide transport	Biological Process	1	11	0.01476
GO:0010182	sugar mediated signaling pathway	Biological Process	5	235	0.0151
GO:0009756	carbohydrate mediated signaling	Biological Process	5	235	0.0151
GO:0098542	defense response to other organism	Biological Process	169	1986	0.01515
GO:0030422	production of siRNA involved in RNA interference	Biological Process	1	224	0.01522
GO:0006984	ER-nucleus signaling pathway	Biological Process	50	381	0.01535
GO:0046482	para-aminobenzoic acid metabolic process	Biological Process	5	83	0.01544
GO:0014070	response to organic cyclic compound	Biological Process	124	1606	0.01566
GO:0015851	nucleobase transport	Biological Process	3	117	0.01582
GO:0008283	cell proliferation	Biological Process	4	495	0.0159
GO:0042554	superoxide anion generation	Biological Process	1	4	0.01599
GO:0006145	purine nucleobase catabolic process	Biological Process	1	4	0.01599
GO:0046110	xanthine metabolic process	Biological Process	1	4	0.01599
GO:0002682	regulation of immune system process	Biological Process	107	881	0.01605
GO:0043067	regulation of programmed cell death	Biological Process	97	818	0.01609
GO:0042178	xenobiotic catabolic process	Biological Process	1	3	0.01609
GO:0010604	positive regulation of macromolecule metabolic process	Biological Process	22	892	0.01611

GO:0048829	root cap development	Biological Process	2	28	0.01613
GO:1903047	mitotic cell cycle process	Biological Process	5	498	0.01619
GO:0032504	multicellular organism reproduction	Biological Process	9	751	0.01642
GO:0009814	defense response, incompatible interaction	Biological Process	117	1057	0.0166
GO:0034470	ncRNA processing	Biological Process	3	520	0.0173
GO:0016310	phosphorylation	Biological Process	142	2328	0.01736
GO:0007231	osmosensory signaling pathway	Biological Process	1	7	0.01756
GO:2000026	regulation of multicellular organismal development	Biological Process	21	1319	0.01768
GO:0009056	catabolic process	Biological Process	107	4089	0.01775
GO:0044267	cellular protein metabolic process	Biological Process	149	5798	0.01781
GO:0009116	nucleoside metabolic process	Biological Process	19	896	0.01814
GO:0009891	positive regulation of biosynthetic process	Biological Process	42	1075	0.01847
GO:0030001	metal ion transport	Biological Process	31	1083	0.01856
GO:0071322	cellular response to carbohydrate stimulus	Biological Process	5	249	0.01863
GO:0043086	negative regulation of catalytic activity	Biological Process	4	280	0.01907
GO:0048645	animal organ formation	Biological Process	2	398	0.01908
GO:0031328	positive regulation of cellular biosynthetic process	Biological Process	22	843	0.01929
GO:0052558	induction by organism of immune response of other organism involved in symbiotic interaction	Biological Process	1	3	0.01953
GO:0052559	induction by symbiont of host immune response	Biological Process	1	3	0.01953
GO:0080185	effector dependent induction by symbiont of host immune response	Biological Process	1	3	0.01953
GO:0060919	auxin influx	Biological Process	1	7	0.01958
GO:0048646	anatomical structure formation involved in morphogenesis	Biological Process	3	584	0.02064
GO:0040034	regulation of development, heterochronic	Biological Process	1	69	0.02064
GO:0046486	glycerolipid metabolic process	Biological Process	12	447	0.02085
GO:1901362	organic cyclic compound biosynthetic process	Biological Process	230	6061	0.02098

GO:0048522	positive regulation of cellular process	Biological Process	38	1427	0.02124
GO:0046039	GTP metabolic process	Biological Process	3	276	0.02126
GO:0048729	tissue morphogenesis	Biological Process	1	10	0.0215
GO:0006396	RNA processing	Biological Process	12	1403	0.02151
GO:0006468	protein phosphorylation	Biological Process	74	1591	0.02154
GO:0010230	alternative respiration	Biological Process	2	2	0.02177
GO:0016052	carbohydrate catabolic process	Biological Process	16	1123	0.02278
GO:0006863	purine nucleobase transport	Biological Process	3	115	0.02283
GO:0048609	multicellular organismal reproductive process	Biological Process	7	716	0.02303
GO:0034220	ion transmembrane transport	Biological Process	15	529	0.02312
GO:0051503	adenine nucleotide transport	Biological Process	1	17	0.02422
GO:0030026	cellular manganese ion homeostasis	Biological Process	1	16	0.02439
GO:1901068	guanosine-containing compound metabolic process	Biological Process	3	290	0.02453
GO:0071705	nitrogen compound transport	Biological Process	80	1366	0.02454
GO:0002237	response to molecule of bacterial origin	Biological Process	20	144	0.025
GO:0000281	mitotic cytokinesis	Biological Process	5	362	0.0255
GO:0000911	cytokinesis by cell plate formation	Biological Process	5	362	0.0255
GO:1902410	mitotic cytokinetic process	Biological Process	5	362	0.0255
GO:0046777	protein autophosphorylation	Biological Process	15	289	0.02581
GO:0010337	regulation of salicylic acid metabolic process	Biological Process	6	22	0.02606
GO:0009753	response to jasmonic acid	Biological Process	96	981	0.02644
GO:1901069	guanosine-containing compound catabolic process	Biological Process	2	270	0.02654
GO:0006184	obsolete GTP catabolic process	Biological Process	2	270	0.02654
GO:0008033	tRNA processing	Biological Process	1	91	0.02658
GO:0010107	potassium ion import	Biological Process	1	11	0.02664
GO:0006638	neutral lipid metabolic process	Biological Process	3	40	0.02671

GO:0006639	acylglycerol metabolic process	Biological Process	3	40	0.02671
GO:0072521	purine-containing compound metabolic process	Biological Process	23	952	0.02725
GO:0005975	carbohydrate metabolic process	Biological Process	71	3346	0.02729
GO:0044281	small molecule metabolic process	Biological Process	176	5596	0.02793
GO:0044728	DNA methylation or demethylation	Biological Process	1	301	0.02832
GO:0006304	DNA modification	Biological Process	1	301	0.02832
GO:0010557	positive regulation of macromolecule biosynthetic process	Biological Process	21	829	0.02885
GO:0072334	UDP-galactose transmembrane transport	Biological Process	1	8	0.02896
GO:0048583	regulation of response to stimulus	Biological Process	132	1564	0.02905
GO:0007264	small GTPase mediated signal transduction	Biological Process	2	232	0.02928
GO:0006305	DNA alkylation	Biological Process	1	298	0.02997
GO:0006306	DNA methylation	Biological Process	1	298	0.02997
GO:0007568	aging	Biological Process	23	320	0.03012
GO:1901616	organic hydroxy compound catabolic process	Biological Process	3	74	0.03018
GO:0061640	cytoskeleton-dependent cytokinesis	Biological Process	9	699	0.03036
GO:0009909	regulation of flower development	Biological Process	6	639	0.03084
GO:0046128	purine ribonucleoside metabolic process	Biological Process	18	835	0.03087
GO:0006163	purine nucleotide metabolic process	Biological Process	21	899	0.03234
GO:0042278	purine nucleoside metabolic process	Biological Process	18	839	0.0324
GO:0009119	ribonucleoside metabolic process	Biological Process	19	868	0.03278
GO:0032506	cytokinetic process	Biological Process	6	370	0.03292
GO:0006680	glucosylceramide catabolic process	Biological Process	3	5	0.03313
GO:0006678	glucosylceramide metabolic process	Biological Process	3	5	0.03313
GO:0042343	indole glucosinolate metabolic process	Biological Process	6	44	0.03342
GO:0031347	regulation of defense response	Biological Process	125	1103	0.0336
GO:0019433	triglyceride catabolic process	Biological Process	1	7	0.03401

GO:0000910	cytokinesis	Biological Process	10	739	0.0345
GO:0010039	response to iron ion	Biological Process	4	92	0.03461
GO:0098662	inorganic cation transmembrane transport	Biological Process	9	325	0.03466
GO:0009627	systemic acquired resistance	Biological Process	103	881	0.03531
GO:0050776	regulation of immune response	Biological Process	105	876	0.03534
GO:0042127	regulation of cell proliferation	Biological Process	1	212	0.03546
GO:0051173	positive regulation of nitrogen compound metabolic process	Biological Process	19	806	0.03565
GO:0023052	signaling	Biological Process	229	3513	0.03601
GO:0010038	response to metal ion	Biological Process	24	1295	0.03698
GO:0048856	anatomical structure development	Biological Process	97	6276	0.03733
GO:0061024	membrane organization	Biological Process	96	1096	0.03796
GO:0051254	positive regulation of RNA metabolic process	Biological Process	18	780	0.03814
GO:1902680	positive regulation of RNA biosynthetic process	Biological Process	18	780	0.03814
GO:0009809	lignin biosynthetic process	Biological Process	9	113	0.03821
GO:0019693	ribose phosphate metabolic process	Biological Process	22	1070	0.03838
GO:0009259	ribonucleotide metabolic process	Biological Process	22	1070	0.03838
GO:0045935	positive regulation of nucleobase-containing compound metabolic process	Biological Process	19	797	0.03839
GO:0006641	triglyceride metabolic process	Biological Process	3	36	0.03839
GO:0048585	negative regulation of response to stimulus	Biological Process	81	715	0.03845
GO:0009744	response to sucrose	Biological Process	19	395	0.03856
GO:0034285	response to disaccharide	Biological Process	19	395	0.03856
GO:1901957	regulation of cutin biosynthetic process	Biological Process	2	4	0.03883
GO:1901959	positive regulation of cutin biosynthetic process	Biological Process	2	4	0.03883
GO:0044700	single organism signaling	Biological Process	229	3512	0.03889
GO:0009612	response to mechanical stimulus	Biological Process	15	110	0.03904
GO:0098655	cation transmembrane transport	Biological Process	10	334	0.03913

GO:0010228	vegetative to reproductive phase transition of meristem	Biological Process	8	808	0.03943
GO:0071281	cellular response to iron ion	Biological Process	2	60	0.03972
GO:0009968	negative regulation of signal transduction	Biological Process	4	123	0.03995
GO:0048523	negative regulation of cellular process	Biological Process	90	1498	0.04038
GO:0000160	phosphorelay signal transduction system	Biological Process	27	304	0.04084
GO:0009749	response to glucose	Biological Process	4	174	0.04319
GO:0006879	cellular iron ion homeostasis	Biological Process	1	22	0.0432
GO:0019438	aromatic compound biosynthetic process	Biological Process	222	5669	0.04335
GO:0009620	response to fungus	Biological Process	113	989	0.04378
GO:0071489	cellular response to red or far red light	Biological Process	8	85	0.04396
GO:0043589	skin morphogenesis	Biological Process	1	3	0.04443
GO:0048730	epidermis morphogenesis	Biological Process	1	3	0.04443
GO:0010073	meristem maintenance	Biological Process	7	548	0.04465
GO:0055086	nucleobase-containing small molecule metabolic process	Biological Process	29	1805	0.04471
GO:0010017	red or far-red light signaling pathway	Biological Process	8	82	0.04517
GO:0055062	phosphate ion homeostasis	Biological Process	6	20	0.04523
GO:0072506	trivalent inorganic anion homeostasis	Biological Process	6	20	0.04523
GO:0051253	negative regulation of RNA metabolic process	Biological Process	28	713	0.04538
GO:0045892	negative regulation of transcription, DNA-templated	Biological Process	28	713	0.04538
GO:1902679	negative regulation of RNA biosynthetic process	Biological Process	28	713	0.04538
GO:0006464	cellular protein modification process	Biological Process	130	3925	0.04544
GO:0036211	protein modification process	Biological Process	130	3925	0.04544
GO:0006865	amino acid transport	Biological Process	43	542	0.04618
GO:0071248	cellular response to metal ion	Biological Process	2	77	0.0463
GO:0010101	post-embryonic root morphogenesis	Biological Process	3	100	0.04633
GO:0010102	lateral root morphogenesis	Biological Process	3	100	0.04633

GO:0030149	sphingolipid catabolic process	Biological Process	3	13	0.0466
GO:0009150	purine ribonucleotide metabolic process	Biological Process	20	834	0.04671
GO:0048232	male gamete generation	Biological Process	1	60	0.04699
GO:0045893	positive regulation of transcription, DNA-templated	Biological Process	18	778	0.04721
GO:0071545	inositol phosphate catabolic process	Biological Process	1	47	0.04747
GO:0046855	inositol phosphate dephosphorylation	Biological Process	1	47	0.04747
GO:0046838	phosphorylated carbohydrate dephosphorylation	Biological Process	1	47	0.04747
GO:0010628	positive regulation of gene expression	Biological Process	18	781	0.04938
GO:0043574	peroxisomal transport	Biological Process	3	155	0.04966
GO:0040007	growth	Biological Process	27	1818	0.04967
GO:0032787	monocarboxylic acid metabolic process	Biological Process	120	2547	0.04985
GO:0009504	cell plate	Cellular Component	1	73	0.0032
GO:0065010	extracellular membrane-bounded organelle	Cellular Component	1	11	0.0152
GO:0070062	extracellular exosome	Cellular Component	1	11	0.0152
GO:0043230	extracellular organelle	Cellular Component	1	11	0.0152
GO:0005886	plasma membrane	Cellular Component	197	5364	0.0173
GO:0009331	glycerol-3-phosphate dehydrogenase complex	Cellular Component	1	6	0.0193
GO:0005783	endoplasmic reticulum	Cellular Component	29	1005	0.0219
GO:0005634	nucleus	Cellular Component	272	11418	0.0226
GO:0015629	actin cytoskeleton	Cellular Component	3	87	0.027
GO:0031225	anchored component of membrane	Cellular Component	8	296	0.0389
GO:0071944	cell periphery	Cellular Component	223	6192	0.0411
GO:0005515	protein binding	Molecular Function	135	4532	5.00E-05
GO:0008810	cellulase activity	Molecular Function	2	16	0.00047
GO:0003700	transcription factor activity, sequence-specific DNA binding	Molecular Function	104	2365	0.00117
GO:0047952	glycerol-3-phosphate dehydrogenase [NAD(P)+] activity	Molecular Function	1	4	0.00142

GO:0042973	glucan endo-1,3-beta-D-glucosidase activity	Molecular Function	1	11	0.00195
GO:0015105	arsenite transmembrane transporter activity	Molecular Function	1	17	0.00256
GO:0015197	peptide transporter activity	Molecular Function	5	44	0.00435
GO:0005525	GTP binding	Molecular Function	4	370	0.00452
GO:0016726	oxidoreductase activity, acting on CH or CH2 groups, NAD or NADP as acceptor	Molecular Function	1	5	0.00563
GO:0004672	protein kinase activity	Molecular Function	81	1508	0.00607
GO:0005345	purine nucleobase transmembrane transporter activity	Molecular Function	2	26	0.00628
GO:0010328	auxin influx transmembrane transporter activity	Molecular Function	1	13	0.00666
GO:0043565	sequence-specific DNA binding	Molecular Function	35	564	0.00704
GO:0004536	deoxyribonuclease activity	Molecular Function	2	24	0.00755
GO:0003968	RNA-directed 5'-3' RNA polymerase activity	Molecular Function	1	12	0.01041
GO:0004520	endodeoxyribonuclease activity	Molecular Function	2	22	0.01135
GO:0097599	xylanase activity	Molecular Function	1	16	0.01198
GO:0020037	heme binding	Molecular Function	14	409	0.01234
GO:0004222	metalloendopeptidase activity	Molecular Function	2	63	0.01521
GO:0034062	5'-3' RNA polymerase activity	Molecular Function	1	106	0.01528
GO:0004385	guanylate kinase activity	Molecular Function	1	3	0.0154
GO:0004854	xanthine dehydrogenase activity	Molecular Function	1	4	0.01588
GO:0019201	nucleotide kinase activity	Molecular Function	1	22	0.01672
GO:0015198	oligopeptide transporter activity	Molecular Function	4	34	0.01716
GO:0004175	endopeptidase activity	Molecular Function	9	370	0.0172
GO:0043225	ATPase-coupled anion transmembrane transporter activity	Molecular Function	1	26	0.01805
GO:0004712	protein serine/threonine/tyrosine kinase activity	Molecular Function	2	82	0.01843
GO:1901149	salicylic acid binding	Molecular Function	1	3	0.01962
GO:0004367	glycerol-3-phosphate dehydrogenase [NAD+] activity	Molecular Function	1	6	0.01975
GO:0016765	transferase activity, transferring alkyl or aryl (other than methyl) groups	Molecular Function	5	157	0.01992

GO:0010285	L,L-diaminopimelate aminotransferase activity	Molecular Function	1	4	0.02017
GO:0005200	structural constituent of cytoskeleton	Molecular Function	1	59	0.02108
GO:0000981	RNA polymerase II transcription factor activity, sequence-specific DNA binding	Molecular Function	2	2	0.02153
GO:0009044	xylan 1,4-beta-xylosidase activity	Molecular Function	1	12	0.02472
GO:0004252	serine-type endopeptidase activity	Molecular Function	1	129	0.03052
GO:0004698	calcium-dependent protein kinase C activity	Molecular Function	1	4	0.03128
GO:0004383	guanylate cyclase activity	Molecular Function	4	10	0.03229
GO:0042802	identical protein binding	Molecular Function	10	380	0.03283
GO:0004348	glucosylceramidase activity	Molecular Function	3	5	0.03323
GO:0003779	actin binding	Molecular Function	4	129	0.03706
GO:0015446	ATPase-coupled arsenite transmembrane transporter activity	Molecular Function	1	11	0.0372
GO:0008490	arsenite secondary active transmembrane transporter activity	Molecular Function	1	11	0.0372
GO:0016740	transferase activity	Molecular Function	184	4838	0.03791
GO:0008061	chitin binding	Molecular Function	6	24	0.03844
GO:0008378	galactosyltransferase activity	Molecular Function	1	54	0.04021
GO:0005509	calcium ion binding	Molecular Function	13	388	0.044
GO:0050897	cobalt ion binding	Molecular Function	2	83	0.04897
