# Supplementary material for: Genome-Wide Analysis of Coding and Long Non-Coding RNAs Involved in Cuticular Wax Biosynthesis in Cabbage (Brassica oleracea L. var. capitata)
Source: Int J Mol Sci. 2019 Jun 10;20(11):2820. doi: 10.3390/ijms20112820 (PMC6600401; doi:10.3390/ijms20112820)
Supplement: Supplementary file 1 [file ijms-20-02820-s001.zip › ijms-505007 supplementary/Supplementary Files/Table S5. Functional enrichment analysis of down-regulated genes in plants exhibiting nwgl phenotype based on KEGG metabolic pathways..pdf]

Table S5. Functional enrichment analysis of down-regulated genes in plants exhibiting *nwgl* phenotype based on KEGG metabolic pathways.

KEGG ID	KEGG pathway	Number of KEGG annotated genes in cluster	Total number of KEGG annotated genes in cabbage whole genome	*P* value	Gene
ko00350	Tyrosine metabolism	7	56	2.23E-07	Bol033528;Bol033662;Bol044773;Kohlrabi_newGene_3371; Kohlrabi_newGene_38225;Kohlrabi_newGene_4041; Kohlrabi_newGene_5485
ko00950	Isoquinoline alkaloid biosynthesis	3	37	0.002985413	Bol033528;Bol033662;Kohlrabi_newGene_4041
ko00966	Glucosinolate biosynthesis	2	11	0.003209026	Bol038222;Bol040365
ko00905	Brassinosteroid biosynthesis	2	14	0.005229516	Bol024487;Bol027773
ko00960	Tropane, piperidine and pyridine alkaloid biosynthesis	3	63	0.013208951	Bol033528;Bol033662;Kohlrabi_newGene_4041
ko00520	Amino sugar and nucleotide sugar metabolism	5	182	0.013919444	Bol005996;Bol018118;Bol029542;Bol040770; Kohlrabi_newGene_38591
ko00500	Starch and sucrose metabolism	6	292	0.026587909	Bol010233;Bol018118;Bol029542;Bol033172;Bol040770; Bol043695
ko00270	Cysteine and methionine metabolism	4	146	0.027593341	Bol009286;Bol011986;Bol033528;Bol033662
ko04075	Plant hormone signal transduction	8	493	0.038148276	Bol013169;Bol015047;Bol020600;Bol026821;Bol027853;Bol032146; Bol044800;Bol045679
