# Supplementary material for: Genome-Wide Analysis of Coding and Long Non-Coding RNAs Involved in Cuticular Wax Biosynthesis in Cabbage (Brassica oleracea L. var. capitata)
Source: Int J Mol Sci. 2019 Jun 10;20(11):2820. doi: 10.3390/ijms20112820 (PMC6600401; doi:10.3390/ijms20112820)
Supplement: Supplementary file 1 [file ijms-20-02820-s001.zip › ijms-505007 supplementary/Supplementary Files/Table S6. Functional enrichment analysis of up-regulated genes in plants exhibiting nwgl phenotype based on KEGG metabolic pathways..pdf]

Table S6. Functional enrichment analysis of up-regulated genes in plants exhibiting *nwgl* phenotype based on KEGG metabolic pathways.

KEGG ID	KEGG pathway	Number of KEGG annotated genes in cluster	Total number of KEGG annotated genes in cabbage whole genome	*P* value	Gene
ko04626	Plant-pathogen interaction	15	290	0.000286146	Bol005163;Bol009159;Bol020355;Bol023852;Bol024879;Bol027148;Bol032054;Bol043072;Bol044444;Kohlrabi_newGene_10749;Kohlrabi_newGene_12206;Kohlrabi_newGene_13177;Kohlrabi_newGene_16047;Kohlrabi_newGene_35732;Kohlrabi_newGene_7259
ko00903	Limonene and pinene degradation	6	63	0.00103535	Bol006366;Bol012237;Bol018619;Bol031073;Bol031074;Bol041268
ko00945	Stilbenoid, diarylheptanoid and gingerol biosynthesis	6	66	0.001323202	Bol006366;Bol012237;Bol018619;Bol031073;Bol031074;Bol041268
ko00511	Other glycan degradation	4	29	0.001870092	Bol015060;Bol019294;Bol028725;Bol031643
ko00073	Cutin, suberine and wax biosynthesis	4	47	0.010869156	Bol025251;Bol025577;Bol035700;Bol036039
ko00940	Phenylpropanoid biosynthesis	10	257	0.020704248	Bol007030;Bol013645;Bol014175;Bol020093;Bol020664;Bol024851;Bol032750;Bol032751;Bol043330;Bol045598
ko00591	Linoleic acid metabolism	2	16	0.034526368	Bol009772;Bol010012
ko00600	Sphingolipid metabolism	3	41	0.039785434	Bol015060;Bol028725;Bol031643
ko00500	Starch and sucrose metabolism	10	292	0.044163094	Bol005944;Bol006046;Bol015876;Bol015932;Bol024851;Bol028526;Bol029539;Bol040412;Bol043330;Bol045598
