# Supplementary material for: Genome-Wide Analysis of Coding and Long Non-Coding RNAs Involved in Cuticular Wax Biosynthesis in Cabbage (Brassica oleracea L. var. capitata)
Source: Int J Mol Sci. 2019 Jun 10;20(11):2820. doi: 10.3390/ijms20112820 (PMC6600401; doi:10.3390/ijms20112820)
Supplement: Supplementary file 1 [file ijms-20-02820-s001.zip › ijms-505007 supplementary/Supplementary Files/Table S9. The cis-regulated target genes of differentially expressed lncRNAs between nwgl and wild-type samples..pdf]

Table S9. The *cis*-regulated target genes of differentially expressed lncRNAs between *nwgl* and wild-type samples.

| LncRNA ID | Target genes |
|---|---|
| MSTRG.42696.7 | Bol022238;Bol022241;Bol022232;Bol022239;Bol022235;Bol022233;Bol022234;Bol022231;Bol022236;Bol022242;Bol022230;Bol022237;Bol022240 |
| MSTRG.71296.1 | Bol021072;Bol021073;Bol021071;Bol022179;Bol022180 |
| MSTRG.84276.1 | Bol035742;Bol035740;Bol035750;Bol035746;Bol035737;Bol035743;Bol035736;Bol035751;Bol035739;Bol035747;Bol035738;Bol035749;Bol035748;Bol035752; Bol035735;Bol035744;Bol035745;Bol035734;Bol035733;Bol035741 |
| MSTRG.81531.2 | Bol035250;Bol035246;Bol035248;Bol035251;Bol035247;Bol035249 |
| MSTRG.81399.22 | Bol007242;Bol007243;Bol007241 |
| MSTRG.84276.6 | Bol035747;Bol035739;Bol035736;Bol035751;Bol035743;Bol035737;Bol035746;Bol035742;Bol035740;Bol035750;Bol035741;Bol035733;Bol035744;Bol035734; Bol035745;Bol035735;Bol035752;Bol035749;Bol035748;Bol035738 |
| MSTRG.79435.1 | Bol019356;Bol019348;Bol019355;Bol019350;Bol012684;Bol012683;Bol019352;Bol019353;Bol019357;Bol019359;Bol012682;Bol012681;Bol019358;Bol019354; Bol019360;Bol019347;Bol019349;Bol019351 |
| MSTRG.52331.1 | Bol019455;Bol019453;Bol019456;Bol019448;Bol019457;Bol019449;Bol019451;Bol013382;Bol013381;Bol019454;Bol013383;Bol019450;Bol019452 |
| MSTRG.79315.2 | Bol019298;Bol019317;Bol019299;Bol019305;Bol019318;Bol019303;Bol019315;Bol019302;Bol019314;Bol019295;Bol019309;Bol019306;Bol019311;Bol019296; Bol019300;Bol019301;Bol019304;Bol019294;Bol019313;Bol019308; Bol019292;Bol019310;Bol019297;Bol019307;Bol019312;Bol019293;Bol019316 |
| MSTRG.67492.6 | Bol033820;Bol033818;Bol033837;Bol033816;Bol033811;Bol033824;Bol033822;Bol033831;Bol033835;Bol033838;Bol033827;Bol033823;Bol033806;Bol033833; Bol033815;Bol033810;Bol033836;Bol033834;Bol033829;Bol033832;Bol033825;Bol033840;Bol033817;Bol033812;Bol033809;Bol033813;Bol033821;Bol033826; Bol033807;Bol033841; Bol033830;Bol033814;Bol033819;Bol033839;Bol033808 |
| MSTRG.56032.1 | Bol045240;Bol044969;Bol045237;Bol045239;Bol045238 |
| MSTRG.86147.2 | Bol043552;Bol043547;Bol043545;Bol043546;Bol043554;Bol043532;Bol043534;Bol043541;Bol043530;Bol043542;Bol043536;Bol043549;Bol043540;Bol043558; Bol043551;Bol043531;Bol043539;Bol043555;Bol043535;Bol043543;Bol043548;Bol043550;Bol043537;Bol043557;Bol043538;Bol043556;Bol043533;Bol043553; Bol043544 |
| MSTRG.78339.1 | Bol032241;Bol032218;Bol032217;Bol032234;Bol032229;Bol032215;Bol032207;Bol032219;Bol032235;Bol032208;Bol032222;Bol032225;Bol032206;Bol032239; Bol032223;Bol032240;Bol032205;Bol032220;Bol032226;Bol032216;Bol032238;Bol032230;Bol032214;Bol032211;Bol032221;Bol032237;Bol032213;Bol032227; |

| | |
|---|---|
| | Bol032212;Bol032236;Bol032232;Bol032209;Bol032204;Bol032228;Bol032233;Bol032231;Bol032210;Bol032224 |
| MSTRG.86575.1 | Bol043766;Bol043741;Bol043748;Bol043783;Bol043749;Bol043785;Bol043754;Bol043759;Bol043775;Bol043781;Bol043755;Bol043758;Bol043752;Bol043780; Bol043762;Bol043760;Bol043746;Bol043768;Bol043786;Bol043787;Bol043745;Bol043756;Bol043788;Bol043751;Bol043774;Bol043782;Bol043772;Bol043747; Bol043753;Bol043738;Bol043742;Bol043784;Bol043773;Bol043764;Bol043770;Bol043767;Bol043765;Bol043777;Bol043771;Bol043779;Bol043776;Bol043757; Bol043761;Bol043743;Bol043739;Bol043750;Bol043740;Bol043769;Bol043778;Bol043744;Bol043763 |
| MSTRG.5278.11 | Bol036594;Bol036586;Bol036587;Bol036592;Bol036593;Bol036589;Bol036591;Bol036590;Bol036595;Bol036588 |
| MSTRG.57462.1 | Bol012393;Bol012396;Bol027253;Bol027252;Bol027250;Bol027251;Bol027249;Bol012394;Bol012395 |
| MSTRG.85996.2 | Bol043465;Bol043459;Bol043462;Bol043470;Bol043477;Bol043466;Bol043472;Bol043456;Bol043475;Bol043461;Bol043455;Bol043476;Bol043468;Bol043474; Bol043471;Bol043467;Bol043453;Bol043473;Bol043478;Bol043458;Bol043457;Bol043463;Bol043460;Bol043454;Bol043469;Bol043464 |
| MSTRG.7109.1 | Bol030990;Bol030995;Bol030989;Bol030992;Bol030997;Bol030999;Bol030998;Bol030994;Bol030996;Bol030993;Bol030991 |
| MSTRG.4233.1 | Bol032794;Bol032792;Bol032791;Bol032793 |
| MSTRG.37172.1 | Bol009936;Bol009941;Bol006757;Bol009937;Bol009939;Bol009945;Bol009938;Bol009944;Bol006754;Bol006755;Bol009943;Bol006756;Bol006753;Bol009942; Bol009940;Bol006752;Bol006751 |
| MSTRG.83647.8 | Bol016848;Bol016847;Bol016839;Bol016844;Bol016842;Bol016850;Bol016849;Bol016841;Bol016840;Bol016851;Bol016852;Bol016846;Bol016845;Bol016843 |
| MSTRG.64296.1 | Bol043137;Bol043141;Bol043138;Bol022274;Bol043146;Bol043143;Bol022276;Bol043139;Bol022273;Bol043144;Bol022275;Bol043140;Bol043136;Bol043145; Bol022278;Bol022271;Bol022269;Bol043142;Bol022270;Bol043135;Bol022272;Bol022277 |
| MSTRG.31955.1 | Bol011693;Bol011688;Bol011690;Bol011695;Bol011689;Bol011687;Bol011694;Bol011691;Bol011685;Bol011686 |
| MSTRG.78518.1 | Bol032336;Bol032345;Bol032331;Bol032346;Bol032318;Bol032329;Bol032333;Bol032324;Bol032316;Bol032338;Bol032335;Bol032334;Bol032339;Bol032342; Bol032317;Bol032327;Bol032323;Bol032321;Bol032330;Bol032347;Bol032343;Bol032328;Bol032332;Bol032341;Bol032320;Bol032325;Bol032322;Bol032340; Bol032337;Bol032315;Bol032344;Bol032326;Bol032319 |
| MSTRG.34028.1 | Bol038625;Bol038623;Bol038622;Bol038624;Bol038628;Bol038626;Bol038629;Bol038619;Bol038621;Bol038627;Bol038620 |
| MSTRG.79413.4 | Bol019344;Bol019345;Bol019355;Bol019350;Bol019339;Bol019356;Bol019338;Bol019348;Bol019343;Bol019342;Bol019358;Bol019354;Bol019346;Bol019341; Bol019347;Bol019349;Bol019351;Bol019352;Bol019353;Bol019357;Bol019340 |
| MSTRG.66579.3 | Bol042182;Bol042162;Bol042169;Bol042173;Bol042171;Bol042172;Bol042180;Bol042181;Bol042149;Bol042154;Bol042178;Bol042153;Bol042151;Bol042152; Bol042166;Bol042175;Bol042176;Bol042160;Bol042163;Bol042168;Bol042179;Bol042177;Bol042157;Bol042158;Bol042170;Bol042156;Bol042148;Bol042155; Bol042183;Bol042164;Bol042174;Bol042161;Bol042165;Bol042184;Bol042159;Bol042167;Bol042150 |

| | |
|---|---|
| MSTRG.85996.1 | Bol043470;Bol043462;Bol043459;Bol043465;Bol043466;Bol043477;Bol043472;Bol043476;Bol043455;Bol043461;Bol043475;Bol043456;Bol043467;Bol043453;Bol043471;Bol043468;Bol043474;Bol043463;Bol043457;Bol043458;Bol043473;Bol043478;Bol043469;Bol043454;Bol043460;Bol043464 |
| MSTRG.83647.4 | Bol016849;Bol016840;Bol016841;Bol016851;Bol016846;Bol016852;Bol016845;Bol016843;Bol016848;Bol016847;Bol016839;Bol016844;Bol016842;Bol016850 |
| MSTRG.31824.4 | Bol004927;Bol004911;Bol004917;Bol004930;Bol004914;Bol004924;Bol004919;Bol004920;Bol004929;Bol004928;Bol004926;Bol004921;Bol004915;Bol004910;Bol004913;Bol004916;Bol004912;Bol004925;Bol004922;Bol004923;Bol004918 |
| MSTRG.80871.2 | Bol012097;Bol012093;Bol012092;Bol012095;Bol012096;Bol012099;Bol012098;Bol012094 |
| MSTRG.81399.23 | Bol007241;Bol007243;Bol007242 |
| MSTRG.5278.1 | Bol036587;Bol036594;Bol036586;Bol036593;Bol036592;Bol036591;Bol036590;Bol036589;Bol036588;Bol036595 |
| MSTRG.13352.1 | Bol039242;Bol039237;Bol039240;Bol039248;Bol039251;Bol039239;Bol039245;Bol039250;Bol039243;Bol039241;Bol039238;Bol039249;Bol039244;Bol039246;Bol039247 |
| MSTRG.35834.2 | Bol027453;Bol027428;Bol027429;Bol027431;Bol027449;Bol027446;Bol027450;Bol027451;Bol027459;Bol027435;Bol027448;Bol027434;Bol027439;Bol027438;Bol027441;Bol027433;Bol027447;Bol027427;Bol027443;Bol027460;Bol027444;Bol027442;Bol027458;Bol027445;Bol027457;Bol027456;Bol027455;Bol027437;Bol027454;Bol027436;Bol027432;Bol027452;Bol027440;Bol027430 |
| MSTRG.79413.2 | Bol019342;Bol019343;Bol019356;Bol019338;Bol019348;Bol019339;Bol019345;Bol019344;Bol019355;Bol019350;Bol019352;Bol019357;Bol019353;Bol019340;Bol019347;Bol019351;Bol019349;Bol019358;Bol019354;Bol019346;Bol019341 |
| MSTRG.79437.1 | Bol019349;Bol019351;Bol019354;Bol019358;Bol019360;Bol019359;Bol012682;Bol012681;Bol012685;Bol019353;Bol019357;Bol019352;Bol012684;Bol012683;Bol019350;Bol019355;Bol019356;Bol019348 |
| MSTRG.11550.10 | Bol018202;Bol018214;Bol018213;Bol018201;Bol018208;Bol018211;Bol018205;Bol018200;Bol018206;Bol018212;Bol018207;Bol018203;Bol018210;Bol018209;Bol018198;Bol018199 |
| MSTRG.20145.1 | Bol028013;Bol028028;Bol028019;Bol028018;Bol028021;Bol028014;Bol028017;Bol028020;Bol028025;Bol028022;Bol028011;Bol028015;Bol028009;Bol028012;Bol028023;Bol028027;Bol028010;Bol028024 |
| MSTRG.34430.3 | Bol014490;Bol014489;Bol014488;Bol014495;Bol014492;Bol014491;Bol014494;Bol014485;Bol014496;Bol014493;Bol014486;Bol014487 |
| MSTRG.82841.1 | Bol038916;Bol038913;Bol038906;Bol038920;Bol038910;Bol038917;Bol038918;Bol038911;Bol038915;Bol038914;Bol038919;Bol038908;Bol038907;Bol038900;Bol038909;Bol038902;Bol038903;Bol038921;Bol038905;Bol038904;Bol038912;Bol038901 |
| MSTRG.81399.19 | Bol007241;Bol007243;Bol007242 |
| MSTRG.81809.7 | Bol029401;Bol029398;Bol029397;Bol029400;Bol029396;Bol029399 |

| | |
|---|---|
| MSTRG.82736.2 | Bol038848;Bol038857;Bol038858;Bol038850;Bol038862;Bol038856;Bol038863;Bol038860;Bol038852;Bol038859;Bol038849;Bol038855;Bol038854;Bol038853;Bol038861;Bol038851 |
| MSTRG.81713.17 | Bol029385;Bol029383;Bol029384;Bol029386;Bol029381;Bol029382 |
| MSTRG.2111.1 | Bol028379;Bol028372;Bol028378;Bol028365;Bol028371;Bol028376;Bol028366;Bol028364;Bol028367;Bol028373;Bol028368;Bol028369;Bol028377;Bol028382;Bol028370;Bol028381;Bol028374;Bol028383;Bol028375;Bol028380 |
| MSTRG.17244.1 | Bol033155;Bol033147;Bol033148;Bol033146;Bol033149;Bol033153;Bol033141;Bol033144;Bol033145;Bol033151;Bol033140;Bol033152;Bol033154;Bol033142;Bol033143;Bol033150 |
| MSTRG.83385.4 | Bol012179;Bol012187;Bol012177;Bol012186;Bol012182;Bol012173;Bol012185;Bol012188;Bol012190;Bol012174;Bol012191;Bol012175;Bol012180;Bol012184;Bol012183;Bol012192;Bol012172;Bol012178;Bol012176;Bol012181;Bol012171;Bol012189 |
| MSTRG.1852.1 | Bol009395;Bol009406;Bol009403;Bol009408;Bol009401;Bol009407;Bol009400;Bol009409;Bol009394;Bol009389;Bol009388;Bol009399;Bol009410;Bol009385;Bol009386;Bol009393;Bol009396;Bol009412;Bol009387;Bol009390;Bol009402;Bol009405;Bol009397;Bol009404;Bol009392;Bol009398;Bol009411;Bol009391;Bol009414;Bol009413 |
| MSTRG.82085.5 | Bol030282;Bol030278;Bol030279;Bol030281;Bol030270;Bol030266;Bol030275;Bol030268;Bol030276;Bol030271;Bol030263;Bol030264;Bol030273;Bol030265;Bol030280;Bol030274;Bol030262;Bol030272;Bol030267;Bol030269 |
| MSTRG.73820.3 | Bol025077;Bol025070;Bol025069;Bol025079;Bol025075;Bol025065;Bol025062;Bol025068;Bol025066;Bol025063;Bol025071;Bol025064;Bol025078;Bol025081;Bol025074;Bol025076;Bol025067;Bol025080;Bol025072;Bol025073 |
| MSTRG.63944.1 | Bol042995;Bol042998;Bol043002;Bol042997;Bol042991;Bol042994;Bol042996;Bol042993;Bol042988;Bol043001;Bol042992;Bol043000;Bol042999;Bol042990;Bol042989 |
| MSTRG.64296.2 | Bol022276;Bol043143;Bol043146;Bol043140;Bol022275;Bol043144;Bol022273;Bol043139;Bol043141;Bol043137;Bol022274;Bol043138;Bol043135;Bol022270;Bol043142;Bol022277;Bol022272;Bol022269;Bol022278;Bol022271;Bol043145;Bol043136 |
| MSTRG.85996.3 | Bol043471;Bol043468;Bol043474;Bol043467;Bol043453;Bol043458;Bol043457;Bol043478;Bol043473;Bol043463;Bol043460;Bol043469;Bol043454;Bol043464;Bol043465;Bol043470;Bol043462;Bol043459;Bol043466;Bol043477;Bol043472;Bol043456;Bol043475;Bol043461;Bol043455;Bol043476 |
| MSTRG.84276.2 | Bol035733;Bol035741;Bol035744;Bol035745;Bol035734;Bol035752;Bol035735;Bol035738;Bol035749;Bol035748;Bol035739;Bol035736;Bol035751;Bol035747;Bol035737;Bol035743;Bol035746;Bol035742;Bol035740;Bol035750 |
| MSTRG.15398.1 | Bol012342;Bol022861;Bol022859;Bol022858;Bol022864;Bol022863;Bol022860;Bol022857;Bol012343;Bol022856;Bol022862 |
| MSTRG.24648.2 | Bol042447;Bol042452;Bol042456;Bol042448;Bol042449;Bol042453;Bol042454;Bol042450;Bol042455 |

| | |
|---|---|
| MSTRG.73820.4 | Bol025076;Bol025074;Bol025073;Bol025072;Bol025080;Bol025067;Bol025071;Bol025081;Bol025078;Bol025064;Bol025063;Bol025066;Bol025069;Bol025070;Bol025077;Bol025062;Bol025068;Bol025065;Bol025079;Bol025075 |
| MSTRG.60301.1 | Bol041797;Bol041795;Bol041803;Bol041806;Bol041799;Bol041789;Bol041791;Bol041798;Bol041800;Bol041796;Bol041793;Bol041790;Bol041801;Bol041807;Bol041788;Bol041792;Bol041805;Bol041802;Bol041794;Bol041804 |
| MSTRG.79413.3 | Bol019355;Bol019350;Bol019344;Bol019345;Bol019339;Bol019348;Bol019356;Bol019338;Bol019342;Bol019343;Bol019346;Bol019341;Bol019358;Bol019354;Bol019347;Bol019349;Bol019351;Bol019340;Bol019352;Bol019357;Bol019353 |
| MSTRG.79413.5 | Bol019350;Bol019355;Bol019344;Bol019345;Bol019339;Bol019348;Bol019338;Bol019356;Bol019342;Bol019343;Bol019341;Bol019346;Bol019354;Bol019358;Bol019349;Bol019351;Bol019347;Bol019340;Bol019357;Bol019353;Bol019352 |
| MSTRG.79055.1 | Bol036185;Bol036171;Bol036193;Bol036190;Bol036177;Bol036188;Bol036176;Bol036181;Bol036170;Bol036175;Bol036169;Bol036180;Bol036189;Bol036172;Bol036173;Bol036187;Bol036191;Bol036174;Bol036178;Bol036186;Bol036179;Bol036182;Bol036192;Bol036194;Bol036184 |
| MSTRG.81809.5 | Bol029401;Bol029398;Bol029397;Bol029400;Bol029396;Bol029399 |
| MSTRG.41437.1 | Bol038017;Bol038009;Bol038015;Bol038010;Bol038008;Bol038018;Bol038007;Bol038014;Bol038013;Bol038011;Bol038012;Bol038016 |
| MSTRG.65060.17 | Bol016976;Bol016971;Bol016968;Bol016964;Bol016972;Bol016969;Bol016974;Bol016965;Bol016975;Bol016955;Bol016967;Bol016957;Bol016961;Bol016966;Bol016960;Bol016958;Bol016956;Bol016962;Bol016970;Bol016959;Bol016963;Bol016973 |
| MSTRG.83911.14 | Bol017266;Bol017270;Bol017268;Bol017267;Bol017261;Bol017271;Bol017262;Bol017260;Bol017269;Bol017273;Bol017264;Bol017263;Bol017272;Bol017265;Bol017259 |
| MSTRG.83634.8 | Bol016858;Bol016849;Bol016851;Bol016852;Bol016861;Bol016848;Bol016847;Bol016860;Bol016859;Bol016862;Bol016856;Bol016855;Bol016863;Bol016865;Bol016853;Bol016857;Bol016854;Bol016864;Bol016850 |
| MSTRG.22347.2 | Bol025650;Bol025655;Bol025646;Bol025654;Bol025652;Bol025642;Bol025643;Bol025648;Bol025656;Bol025640;Bol025641;Bol025639;Bol025647;Bol025649;Bol025644;Bol025638;Bol025651;Bol025653;Bol025645 |
| MSTRG.24730.2 | Bol042492;Bol042489;Bol042488;Bol042474;Bol042493;Bol042487;Bol042480;Bol042475;Bol042486;Bol042476;Bol042490;Bol042473;Bol042485;Bol042472;Bol042478;Bol042482;Bol042481;Bol042483;Bol042479;Bol042477;Bol042491;Bol042484 |
| MSTRG.31824.2 | Bol004922;Bol004923;Bol004918;Bol004910;Bol004915;Bol004921;Bol004912;Bol004925;Bol004916;Bol004913;Bol004920;Bol004926;Bol004928;Bol004929;Bol004917;Bol004911;Bol004927;Bol004919;Bol004924;Bol004914;Bol004930 |
| MSTRG.34603.3 | Bol017575;Bol008378;Bol008379;Bol017566;Bol017569;Bol017571;Bol008381;Bol008383;Bol017568;Bol017570;Bol008382;Bol008384;Bol017573;Bol017574;Bol017567;Bol008380;Bol017572 |

| | |
|---|---|
| MSTRG.82076.6 | Bol030270;Bol030266;Bol030275;Bol030277;Bol030268;Bol030278;Bol030279;Bol030274;Bol030262;Bol030272;Bol030267;Bol030269;Bol030271;Bol030276;Bol030263;Bol030265;Bol030264 |
| MSTRG.16553.2 | Bol020064;Bol020059;Bol020065;Bol020063;Bol020055;Bol020049;Bol020053;Bol020061;Bol020056;Bol020050;Bol020060;Bol020054;Bol020057;Bol020051;Bol020058;Bol020052;Bol020062 |
| MSTRG.13352.2 | Bol039240;Bol039237;Bol039242;Bol039239;Bol039248;Bol039251;Bol039245;Bol039246;Bol039247;Bol039244;Bol039249;Bol039250;Bol039243;Bol039241;Bol039238 |
| MSTRG.72680.4 | Bol008709;Bol008699;Bol008702;Bol008697;Bol008704;Bol008703;Bol008705;Bol008708;Bol008710;Bol008706;Bol008713;Bol008700;Bol008696;Bol008712;Bol008701;Bol008711;Bol008698;Bol008707 |
| MSTRG.79413.1 | Bol019339;Bol019345;Bol019344;Bol019355;Bol019350;Bol019343;Bol019342;Bol019356;Bol019338;Bol019348;Bol019347;Bol019351;Bol019349;Bol019358;Bol019354;Bol019346;Bol019341;Bol019352;Bol019357;Bol019353;Bol019340 |
| MSTRG.81399.7 | Bol007241;Bol007243;Bol007242 |
| MSTRG.84152.1 | Bol035701;Bol035695;Bol035703;Bol035693;Bol035702;Bol035700;Bol035699;Bol035698;Bol035696;Bol035694;Bol035697;Bol035704 |
| MSTRG.72750.2 | Bol008682;Bol008687;Bol008686;Bol008685;Bol008683;Bol008680;Bol008678;Bol008679;Bol008684;Bol008677;Bol008681;Bol008688;Bol008676;Bol008690;Bol008689 |
| MSTRG.79275.1 | Bol019275;Bol019306;Bol019272;Bol019300;Bol019301;Bol019294;Bol019308;Bol019277;Bol019283;Bol019297;Bol019310;Bol019291;Bol019307;Bol019274;Bol019293;Bol019271;Bol019298;Bol019279;Bol019286;Bol019285;Bol019302;Bol019295;Bol019309;Bol019282;Bol019311;Bol019276;Bol019296;Bol019290;Bol019304;Bol019284;Bol019292;Bol019288;Bol019312;Bol019273;Bol019289;Bol019299;Bol019270;Bol019305;Bol019281;Bol019303;Bol019287;Bol019278;Bol019280 |
| MSTRG.15679.1 | Bol022779;Bol020102;Bol020098;Bol022778;Bol020100;Bol020096;Bol022775;Bol020104;Bol020101;Bol020099;Bol020097;Bol022781;Bol020103;Bol022776;Bol022777;Bol020105;Bol022780;Bol022774 |
| MSTRG.5278.3 | Bol036595;Bol036588;Bol036589;Bol036590;Bol036591;Bol036593;Bol036592;Bol036586;Bol036594;Bol036587 |
| MSTRG.47267.1 | Bol009322;Bol009310;Bol009321;Bol009320;Bol009324;Bol009316;Bol009329;Bol009313;Bol009330;Bol009319;Bol009317;Bol009327;Bol009314;Bol009325;Bol009323;Bol009326;Bol009312;Bol009315;Bol009318;Bol009328;Bol009309;Bol009311 |
| MSTRG.28431.1 | Bol016090;Bol016081;Bol016082;Bol016099;Bol016093;Bol016095;Bol016088;Bol016091;Bol016078;Bol016086;Bol016098;Bol016080;Bol016096;Bol016085;Bol016084;Bol016094;Bol016097;Bol016083;Bol016079;Bol016089;Bol016092;Bol016087 |
| MSTRG.11550.5 | Bol018199;Bol018209;Bol018198;Bol018203;Bol018210;Bol018207;Bol018206;Bol018212;Bol018211;Bol018200;Bol018205;Bol018201;Bol018208;Bol018213; |

| | |
|---|---|
| | Bol018214;Bol018202 |
| MSTRG.49823.1 | Bol032845;Bol032831;Bol032838;Bol032832;Bol032834;Bol032842;Bol032835;Bol032830;Bol032841;Bol032839;Bol032840;Bol032837;Bol032844;Bol032833;Bol032836;Bol032843 |
| MSTRG.26591.1 | Bol041399;Bol041398;Bol041400 |
| MSTRG.16044.1 | Bol014848;Bol014853;Bol014839;Bol014841;Bol014851;Bol014850;Bol014855;Bol014844;Bol014840;Bol014842;Bol014856;Bol014845;Bol014846;Bol014847;Bol014849;Bol014854;Bol014852;Bol014843 |
| MSTRG.85996.4 | Bol043466;Bol043477;Bol043465;Bol043470;Bol043462;Bol043459;Bol043456;Bol043475;Bol043461;Bol043455;Bol043476;Bol043472;Bol043458;Bol043457;Bol043478;Bol043473;Bol043463;Bol043471;Bol043474;Bol043468;Bol043467;Bol043453;Bol043464;Bol043460;Bol043469;Bol043454 |
| MSTRG.49742.1 | Bol032864;Bol032866;Bol032858;Bol032862;Bol032863;Bol032867;Bol032857;Bol032860;Bol032856;Bol032861;Bol032865;Bol032859 |
| MSTRG.56081.4 | Bol044967;Bol044966;Bol044964;Bol044965;Bol045233;Bol044968 |
| MSTRG.81713.9 | Bol029383;Bol029385;Bol029381;Bol029382;Bol029384;Bol029386 |
| MSTRG.83634.9 | Bol016848;Bol016861;Bol016847;Bol016860;Bol016859;Bol016862;Bol016849;Bol016858;Bol016851;Bol016852;Bol016853;Bol016857;Bol016864;Bol016854;Bol016850;Bol016856;Bol016855;Bol016863;Bol016865 |
| MSTRG.17989.1 | Bol016451;Bol016453;Bol016450;Bol016443;Bol016434;Bol016433;Bol016447;Bol016438;Bol016441;Bol016446;Bol016437;Bol016439;Bol016435;Bol016436;Bol016442;Bol016440;Bol016431;Bol016444;Bol016448;Bol016452;Bol016432;Bol016449 |
| MSTRG.22397.1 | Bol025673;Bol025672;Bol025674;Bol025685;Bol025675;Bol025683;Bol025680;Bol025684;Bol025679;Bol025677;Bol025681;Bol025678;Bol025676;Bol025682 |
| MSTRG.56258.9 | Bol045224;Bol044949;Bol044950;Bol045223;Bol044951 |
| MSTRG.81713.24 | Bol029385;Bol029383;Bol029384;Bol029386;Bol029382;Bol029381 |
| MSTRG.4368.1 | Bol021935;Bol021933;Bol021930;Bol021929;Bol021932;Bol021931;Bol021928;Bol021934;Bol021936 |
| MSTRG.82076.5 | Bol030278;Bol030279;Bol030268;Bol030277;Bol030275;Bol030270;Bol030266;Bol030263;Bol030264;Bol030265;Bol030276;Bol030271;Bol030269;Bol030272;Bol030267;Bol030262;Bol030274 |
| MSTRG.58820.1 | Bol041585;Bol041588;Bol041584;Bol041587;Bol041586;Bol041589 |
| MSTRG.64884.1 | Bol017065;Bol017047;Bol017059;Bol017044;Bol017045;Bol017063;Bol017053;Bol017066;Bol017046;Bol017064;Bol017056;Bol017042;Bol017052;Bol017060;Bol017062;Bol017061;Bol017057;Bol017058;Bol017051;Bol017043;Bol017049;Bol017055;Bol017050;Bol017054;Bol017041;Bol017048 |
| MSTRG.12596.3 | Bol034897;Bol034898;Bol034902;Bol034901;Bol034899;Bol034903;Bol034900;Bol034894;Bol034895 |
| MSTRG.3278.2 | Bol039599;Bol039603;Bol039606;Bol039602;Bol039607;Bol039604;Bol039590;Bol039594;Bol039601;Bol039591;Bol039609;Bol039595;Bol039600;Bol039592; |

| | |
|---|---|
| | Bol039605;Bol039596;Bol039593;Bol039598;Bol039608;Bol039597 |
| MSTRG.41422.1 | Bol038004;Bol038011;Bol038006;Bol038005;Bol038009;Bol038010;Bol038003;Bol038008;Bol038007 |
| MSTRG.5114.2 | Bol036554;Bol036555;Bol036549;Bol036550;Bol036556;Bol036546;Bol036547;Bol036552;Bol036548;Bol036553;Bol036551 |
| MSTRG.81399.16 | Bol007242;Bol007243;Bol007241 |
| MSTRG.83634.13 | Bol016854;Bol016850;Bol016853;Bol016857;Bol016863;Bol016856;Bol016855;Bol016862;Bol016848;Bol016861;Bol016847;Bol016860;Bol016859;Bol016851;Bol016852;Bol016858;Bol016849 |
| MSTRG.83647.9 | Bol016845;Bol016843;Bol016852;Bol016846;Bol016851;Bol016841;Bol016840;Bol016849;Bol016850;Bol016842;Bol016844;Bol016839;Bol016847;Bol016848 |
| MSTRG.85996.5 | Bol043476;Bol043455;Bol043456;Bol043475;Bol043461;Bol043472;Bol043466;Bol043477;Bol043470;Bol043462;Bol043459;Bol043465;Bol043464;Bol043469;Bol043454;Bol043460;Bol043463;Bol043458;Bol043457;Bol043473;Bol043478;Bol043467;Bol043453;Bol043471;Bol043468;Bol043474 |
| MSTRG.34912.2 | Bol006712;Bol006706;Bol006710;Bol006705;Bol017656;Bol006708;Bol006703;Bol006714;Bol006711;Bol006704;Bol006707;Bol006713;Bol006709;Bol017657 |
| MSTRG.22003.1 | Bol015040;Bol015045;Bol015036;Bol015050;Bol015035;Bol015041;Bol015048;Bol015034;Bol015039;Bol015037;Bol015054;Bol015043;Bol015047;Bol015044;Bol015052;Bol015051;Bol015046;Bol015049;Bol015053;Bol015033;Bol015038 |
| MSTRG.59880.4 | Bol031849;Bol031852;Bol031851;Bol031854;Bol031845;Bol031855;Bol031853;Bol031850;Bol031846;Bol031847 |
| MSTRG.6596.2 | Bol023243;Bol023242;Bol023244;Bol023237;Bol023248;Bol023246;Bol023239;Bol023241;Bol023247;Bol023245;Bol023236;Bol023238;Bol023240;Bol023235;Bol023249 |
| MSTRG.81399.6 | Bol007241;Bol007243;Bol007242 |
| MSTRG.1896.1 | Bol009383;Bol009387;Bol009369;Bol009390;Bol009365;Bol009382;Bol009377;Bol009367;Bol009384;Bol009391;Bol009379;Bol009371;Bol009392;Bol009374;Bol009376;Bol009366;Bol009381;Bol009375;Bol009370;Bol009364;Bol009388;Bol009389;Bol009380;Bol009393;Bol009372;Bol009373;Bol009368;Bol009386;Bol009385;Bol009378 |
| MSTRG.82076.4 | Bol030278;Bol030279;Bol030268;Bol030277;Bol030275;Bol030270;Bol030266;Bol030263;Bol030264;Bol030265;Bol030271;Bol030276;Bol030269;Bol030272;Bol030267;Bol030262;Bol030274 |
| MSTRG.31824.3 | Bol004918;Bol004923;Bol004922;Bol004916;Bol004912;Bol004925;Bol004913;Bol004910;Bol004921;Bol004915;Bol004926;Bol004928;Bol004929;Bol004920;Bol004924;Bol004919;Bol004930;Bol004914;Bol004911;Bol004917;Bol004927 |
| MSTRG.5278.2 | Bol036593;Bol036592;Bol036587;Bol036594;Bol036586;Bol036588;Bol036595;Bol036591;Bol036590;Bol036589 |
| MSTRG.16064.2 | Bol014828;Bol014841;Bol014824;Bol014834;Bol014839;Bol014836;Bol014840;Bol014825;Bol014831;Bol014844;Bol014829;Bol014838;Bol014832;Bol014833;Bol014845;Bol014835;Bol014842;Bol014843;Bol014830;Bol014827;Bol014837;Bol014847;Bol014826;Bol014846 |

| | |
|---|---|
| MSTRG.15660.1 | Bol022777;Bol022785;Bol022775;Bol022781;Bol022774;Bol022783;Bol022776;Bol022782;Bol022779;Bol022784;Bol022786;Bol022778 |
| MSTRG.14550.1 | Bol016565;Bol016562;Bol016563;Bol016570;Bol016568;Bol016561;Bol016571;Bol016564;Bol016560;Bol016566;Bol016569;Bol016567 |
| MSTRG.5176.1 | Bol036567;Bol036564;Bol036561;Bol036565;Bol036563;Bol036569;Bol036570;Bol036568;Bol036566;Bol036562 |
| MSTRG.6244.5 | Bol028750;Bol028738;Bol028747;Bol028740;Bol028741;Bol028751;Bol028743;Bol028745;Bol028742;Bol028749;Bol028748;Bol028744;Bol028746;Bol028739 |
| MSTRG.71533.1 | Bol022058;Bol022082;Bol022081;Bol022061;Bol022057;Bol022055;Bol022085;Bol022075;Bol022080;Bol022073;Bol022056;Bol022078;Bol022079;Bol022077; Bol022084;Bol022067;Bol022062;Bol022064;Bol022076;Bol022074;Bol022068;Bol022060;Bol022066;Bol022072;Bol022065;Bol022059;Bol022083;Bol022063 |
| MSTRG.74022.1 | Bol025151;Bol025141;Bol025149;Bol025139;Bol025167;Bol025154;Bol025148;Bol025158;Bol025136;Bol025164;Bol025165;Bol025159;Bol025143;Bol025161; Bol025157;Bol025142;Bol025145;Bol025138;Bol025147;Bol025137;Bol025150;Bol025140;Bol025153;Bol025156;Bol025152;Bol025146;Bol025144;Bol025166; Bol025155;Bol025168;Bol025163;Bol025162 |
| MSTRG.86432.1 | Bol043676;Bol043682;Bol043688;Bol043680;Bol043687;Bol043679;Bol043686;Bol043689;Bol043693;Bol043681;Bol043684;Bol043678;Bol043675;Bol043690; Bol043691;Bol043692;Bol043685;Bol043677;Bol043683 |
| MSTRG.21778.6 | Bol029603;Bol029608;Bol029613;Bol029615;Bol029607;Bol029609;Bol029611;Bol029616;Bol029612;Bol029604;Bol029601;Bol029614;Bol029602;Bol029600; Bol029605;Bol029610;Bol029617;Bol029606 |
| MSTRG.6666.5 | Bol023225;Bol023215;Bol023223;Bol023214;Bol023220;Bol023217;Bol023216;Bol023224;Bol023219;Bol023221;Bol023213;Bol023222;Bol023218 |
| MSTRG.83634.10 | Bol016850;Bol016854;Bol016857;Bol016853;Bol016863;Bol016855;Bol016856;Bol016862;Bol016859;Bol016860;Bol016847;Bol016848;Bol016861;Bol016852; Bol016851;Bol016858;Bol016849 |
| MSTRG.63786.1 | Bol042941;Bol042940;Bol042937;Bol042938;Bol042919;Bol042923;Bol042924;Bol042930;Bol042922;Bol042939;Bol042936;Bol042921;Bol042925;Bol042934; Bol042932;Bol042931;Bol042928;Bol042926;Bol042929;Bol042935;Bol042942;Bol042927;Bol042920 |
| MSTRG.38819.7 | Bol037768;Bol037790;Bol037774;Bol037770;Bol037772;Bol037773;Bol037788;Bol037782;Bol037777;Bol037775;Bol037792;Bol037779;Bol037771;Bol037794; Bol037784;Bol037787;Bol037780;Bol037778;Bol037793;Bol037789;Bol037786;Bol037769;Bol037785;Bol037781;Bol037776;Bol037791;Bol037783 |
| MSTRG.47429.1 | Bol005908;Bol005904;Bol005902;Bol005913;Bol009268;Bol005906;Bol005912;Bol005910;Bol005907;Bol005903;Bol005905;Bol005909;Bol005911 |
| MSTRG.14102.3 | Bol020850;Bol020847;Bol020849;Bol020848 |
| MSTRG.85727.9 | Bol030338;Bol030336;Bol030335;Bol030332;Bol030340;Bol030339;Bol030333;Bol030334;Bol030341;Bol030337 |
| MSTRG.85996.6 | Bol043475;Bol043461;Bol043456;Bol043476;Bol043455;Bol043472;Bol043466;Bol043477;Bol043465;Bol043470;Bol043462;Bol043459;Bol043464;Bol043460; Bol043469;Bol043454;Bol043457;Bol043458;Bol043473;Bol043478;Bol043463;Bol043471;Bol043474;Bol043468;Bol043453;Bol043467 |
| MSTRG.81713.23 | Bol029384;Bol029386;Bol029382;Bol029381;Bol029385;Bol029383 |

| | |
|---|---|
| MSTRG.36277.3 | Bol044171;Bol044169;Bol044168;Bol044173;Bol044172;Bol044175;Bol044178;Bol044179;Bol044170;Bol044177;Bol044174;Bol044180;Bol044176;Bol044167 |
| MSTRG.6244.7 | Bol028745;Bol028742;Bol028749;Bol028748;Bol028744;Bol028739;Bol028746;Bol028750;Bol028738;Bol028747;Bol028740;Bol028741;Bol028743;Bol028751 |
| MSTRG.76018.3 | Bol044770;Bol044765;Bol045844;Bol045861;Bol045851;Bol045853;Bol045848;Bol045847;Bol044768;Bol045855;Bol044778;Bol044773;Bol045852;Bol044764; Bol044767;Bol044775;Bol045845;Bol044779;Bol045854;Bol045862;Bol044766;Bol045846;Bol044771;Bol044777;Bol045850;Bol045856;Bol045849;Bol045859; Bol044761;Bol044763;Bol045860;Bol044762;Bol044776;Bol045863;Bol045857;Bol044769;Bol044774;Bol044780;Bol044760;Bol044772;Bol045858 |
| MSTRG.49534.1 | Bol032928;Bol032930;Bol032939;Bol032937;Bol032932;Bol032927;Bol032931;Bol032933;Bol032942;Bol032929;Bol032941;Bol032938;Bol032943;Bol032940; Bol032934;Bol032935;Bol032936 |
| MSTRG.72772.1 | Bol007411;Bol008678;Bol008666;Bol007409;Bol008671;Bol008663;Bol008664;Bol008676;Bol008670;Bol008667;Bol008669;Bol008679;Bol007410;Bol008668; Bol008672;Bol007408;Bol007412;Bol008673;Bol008665;Bol007413;Bol008675;Bol008674;Bol008677 |
| MSTRG.85475.2 | Bol030428;Bol030432;Bol030429;Bol030409;Bol030412;Bol030417;Bol030424;Bol030430;Bol030415;Bol030410;Bol030411;Bol030413;Bol030423;Bol030418; Bol030426;Bol030416;Bol030427;Bol030425;Bol030414;Bol030419;Bol030421;Bol030431;Bol030420;Bol030422 |
| MSTRG.48667.1 | Bol027741;Bol027759;Bol027746;Bol026086;Bol027745;Bol026084;Bol026087;Bol027757;Bol027743;Bol027754;Bol026094;Bol026089;Bol027753;Bol027752; Bol026095;Bol026085;Bol027742;Bol026091;Bol026092;Bol026082;Bol027758;Bol027750;Bol026096;Bol026097;Bol026083;Bol027751;Bol027748;Bol027744; Bol026090;Bol027747;Bol027756;Bol026093;Bol026088;Bol027760;Bol027755;Bol027749;Bol027740 |
| MSTRG.5278.7 | Bol036586;Bol036594;Bol036587;Bol036593;Bol036592;Bol036589;Bol036590;Bol036591;Bol036595;Bol036588 |
| MSTRG.79974.1 | Bol018928;Bol018940;Bol018936;Bol018929;Bol018933;Bol018942;Bol018931;Bol018932;Bol018934;Bol018943;Bol018944;Bol018930;Bol018937;Bol018941; Bol018935;Bol018939;Bol018938 |
| MSTRG.81399.5 | Bol007242;Bol007243;Bol007241 |
| MSTRG.81713.18 | Bol029386;Bol029384;Bol029381;Bol029382;Bol029385;Bol029383 |
| MSTRG.6741.1 | Bol018781;Bol018777;Bol018780;Bol018786;Bol018779;Bol018782;Bol018778;Bol018787;Bol018776;Bol018790;Bol018788;Bol018784;Bol018783;Bol018789; Bol018791;Bol018785 |
